# Biological *J*‐Coupling Spectroscopy at Low Magnetic Field

**DOI:** 10.1002/smsc.202500268

**Published:** 2025-07-31

**Authors:** Gonzalo G. Rodriguez, Charlotte von Petersdorff‐Campen, Sergey Korchak, Oscar Sucre, Maria D. Santi, Josef Elsasser, Ruhuai Mei, Lisa M. Fries, Jan Felger, Andrea Markus, Frauke Alves, Stefan Glöggler

**Affiliations:** ^1^ NMR Signal Enhancement Group Max Planck Institute for Multidisciplinary Sciences Am Fassberg 11 37077 Göttingen Germany; ^2^ Center for Biostructural Imaging of Neurodegeneration University Medical Center Göttingen Von‐Siebold‐Str. 3A 37075 Göttingen Germany; ^3^ Translational Molecular Imaging Max Planck Institute for Multidisciplinary Sciences Hermann Rein Str. 3 37075 Göttingen Germany; ^4^ Institute for Clinical and Interventional Radiology University Medical Center Göttingen Robert‐Koch‐Str. 40 37075 Göttingen Germany; ^5^ Clinic of Hematology and Medical Oncology University Medical Center Göttingen Robert‐Koch‐Str. 40 37075 Göttingen Germany

**Keywords:** cellular metabolism, hyperpolarization, *J* ‐coupling, low-field MRI, magnetic resonance imaging, parahydrogen, pyruvate

## Abstract

Nuclear magnetic resonance (NMR) spectroscopy is a powerful tool for investigating biological systems. In particular, low‐field NMR offers advantages for studying cellular metabolism in their native environment. This is especially relevant for in vitro studies, where low‐field NMR can be used to analyze biological samples with reduced equipment size and cost, potentially enabling high‐throughput benchtop research. However, the study of cellular metabolism at low, milli‐Tesla, magnetic fields remains an unsolved challenge due to reduced chemical shifts and dominant spin‐spin couplings (*J*‐couplings) that complicate spectral analysis. Herein, this problem is tackled by combining *J*‐coupling spectroscopy and parahydrogen‐induced polarization with a multinuclear scanner built in‐house. The results demonstrate the ability to resolve pyruvate metabolism of cancer cells in regular NMR tubes at milli‐Tesla fields using [2‐^13^C]pyruvate. This work concludes that low‐field biological *J*‐coupling spectroscopy can be a valuable tool for studying cellular metabolism, enabling new insights into biological systems.

## Graphical abstract

The strong *J*(C,H)‐coupling from [2‐^13^C] pyruvate is exploited to measure the pyruvate‐to‐lactate conversion in cancer cells at low magnetic fields. This is combined with a parahydrogen‐induced polarizationprotocol optimized to provide a biocompatible solution with 20 000‐fold signal enhancement. Finally, the pyruvate and lactate spectra in murine Panc02 cancer cells are measured, and the pyruvate concentration is calculated.

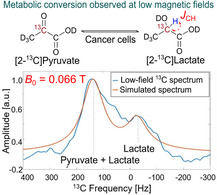

## 1 Introduction

Nuclear magnetic resonance (NMR) spectroscopy has seen a continuous push toward higher magnetic field strengths to enhance spectral resolution and sensitivity. However, there are numerous scenarios where low magnetic fields offer distinct advantages, such as in benchtop and educational instruments,^[^ ^1^, ^2^ ^]^ spectroscopy in the presence of ferromagnetic and paramagnetic substances,^[^ ^3^ ^]^ and optically detected NMR with nitrogen vacancies as sensors.^[^ ^4^ ^]^ Furthermore, low‐field NMR spectroscopy holds great potential for studying the metabolism of biological systems in their native environments, enabling to gain insights into molecular behaviors that are not always accessible with high‐field systems. This is especially relevant for in vitro studies, where low‐field NMR can be used to analyze biological samples with reduced equipment size and cost, making it more accessible for laboratory/benchtop research. Additionally, low‐field biological NMR spectroscopy could pave the way for innovative in vivo applications, such as portable devices for imaging metabolism and spectroscopy in living organisms, which could revolutionize diagnostics and monitoring diseases in clinical settings.

Despite these promising opportunities, low‐field NMR spectroscopy faces challenges rooted in the fundamental physics of magnetic resonance. As magnetic field strength decreases, spin‐spin couplings (*J*‐couplings) become dominant over chemical shifts, leading to increasingly complex spectra that deviate from the simple rules of first‐order perturbation theory.^[^ ^5^, ^6^, ^7^ ^]^ At low fields, this can result in the coalescence of spectral lines, reducing the ability to extract structural information or differentiate metabolites. A solution for this problem is to use substrates containing a spin‐1/2 heteronucleus, such as ^13^C, or ^15^N, which interacts with proton spins at low‐field to break magnetic near‐equivalence and produce a distinctive *J*‐coupling spectrum.^[^ ^5^ ^]^ Instead of being separated by chemical shifts, the spectral lines reflect sums, differences, and multiples of the *J*‐coupling strengths among spins, which are unique to each substance. However, the requirement of a coupled heteronucleus imposes a substantial limitation to the study of biological samples given that the low natural abundance and gyromagnetic ratio of ^13^C result in more than a hundred‐fold disadvantage in signal. Remarkably, this low sensitivity can be compensated by the use of hyperpolarization (HP) techniques.

HP can enhance NMR signals over 10 000 times,^[^ ^8^ ^]^ thereby enabling the detection of low‐concentration species, including metabolites internalized by cancer cells.^[^ ^9^ ^]^ Moreover, as the signal enhancement of HP contrast agents is independent of the detection magnetic field (*B*_0_), the signal‐to‐noise ratio (SNR) difference between high‐field and low‐field systems can be minimal when using HP. Thus, in HP experiments, the SNR dependence with the magnetic field can be reduced from *ω*_*0*_^7/4^, as in standard ^1^H NMR,^[^ ^10^ ^]^ to *ω*_*0*_^1/4^ in the coil‐noise‐dominance regime,^[^ ^11^ ^]^ where ω_0_ represents the resonance frequency.

Two primary HP techniques have been developed for real‐time detection of metabolism: dissolution dynamic nuclear polarization (d‐DNP)^[^ ^12^ ^]^ and parahydrogen‐induced polarization (PHIP).^[^ ^13^ ^]^ The d‐DNP method relies on electron polarization as the source of HP, involving the polarization of nuclear spins in the solid state by DNP at low temperatures (1–2 K) and high magnetic fields (3–7 T). This method has demonstrated versatility, enabling the HP of a large variety of compounds.^[^ ^14^ ^]^ However, the requirement of polarizing the electrons results in a low throughput and the need of complex and expensive hardware that limits its implementation for real‐time in vitro benchtop studies.

In contrast, PHIP is a chemically based HP technique that utilizes parahydrogen (*p*H_2_) singlet spin order as source of HP.^[^ ^13^ ^]^ PHIP offers several advantages, including low cost, low instrumentation requirements, high throughput, and scalable production of HP agents in the liquid state.^[^ ^15^ ^]^ Currently, there are two main PHIP methods to hyperpolarize metabolites such as pyruvate: PHIP by side arm hydrogenation (PHIP‐SAH) and Signal Amplification by Reversible Exchange (SABRE). In SABRE, a substrate molecule is attached to a catalyst, which is typically a transition metal complex.^[^ ^16^, ^17^ ^]^ The catalyst is designed to facilitate reversible ligand exchange, where hydrogen atoms stemming from *p*H_2_ and substrates function as ligands. Once both are coordinating the same metal center of the catalyst, spin polarization is transferred from the hydrides to the substrate. Further ligand exchange leads to the release of hydrogen and polarized substrate, during which the vacant coordination sites are filled with new *p*H_2_ and substrates. In PHIP‐SAH, a precursor molecule formed by an unsaturated moiety linked to the substrate of interest is hyperpolarized by PHIP, and upon hydrolysis by base injection, the hyperpolarized metabolite is retained.^[^ ^18^ ^]^ Heteronuclear polarization transfer from parahydrogen is achieved through magnetic field cycling or pulsed NMR methods. Currently, PHIP‐SAH is the more developed PHIP technology and provides higher polarizations values for biologically compatible substrates.^[^ ^19^ ^]^ Furthermore, PHIP‐SAH, and, more recently, SABRE protocols have reached enough signal enhancements and the maturity to even image tumor metabolism in vivo.^[^ ^20^, ^21^ ^]^ More importantly, it was demonstrated that PHIP‐SAH can provide in vivo metabolic results that are comparable with the obtained with the state of the art d‐DNP polarizers.^[^ ^22^ ^]^

Among the HP substrates, pyruvate is of particularly high interest due to the key role of the pyruvate‐to‐lactate conversion in biological systems, and specifically the Warburg effect highlighted in cancer cells.^[^ ^23^ ^]^ Furthermore, the use of PHIP‐SAH [1‐^13^C]pyruvate was recently demonstrated for studying cell metabolism at high magnetic fields (7 T).^[^ ^24^, ^25^ ^]^ At low magnetic fields (*B*_0_ < 1 T), however, the study of cellular metabolism remains an unsolved problem, with no reported studies on in vitro metabolism and one synthetic enzymatic reaction in the nano‐Tesla regime^[^ ^26^ ^]^ and one in the milli‐Tesla regime.^[^ ^27^ ^]^

**Figure 1:**
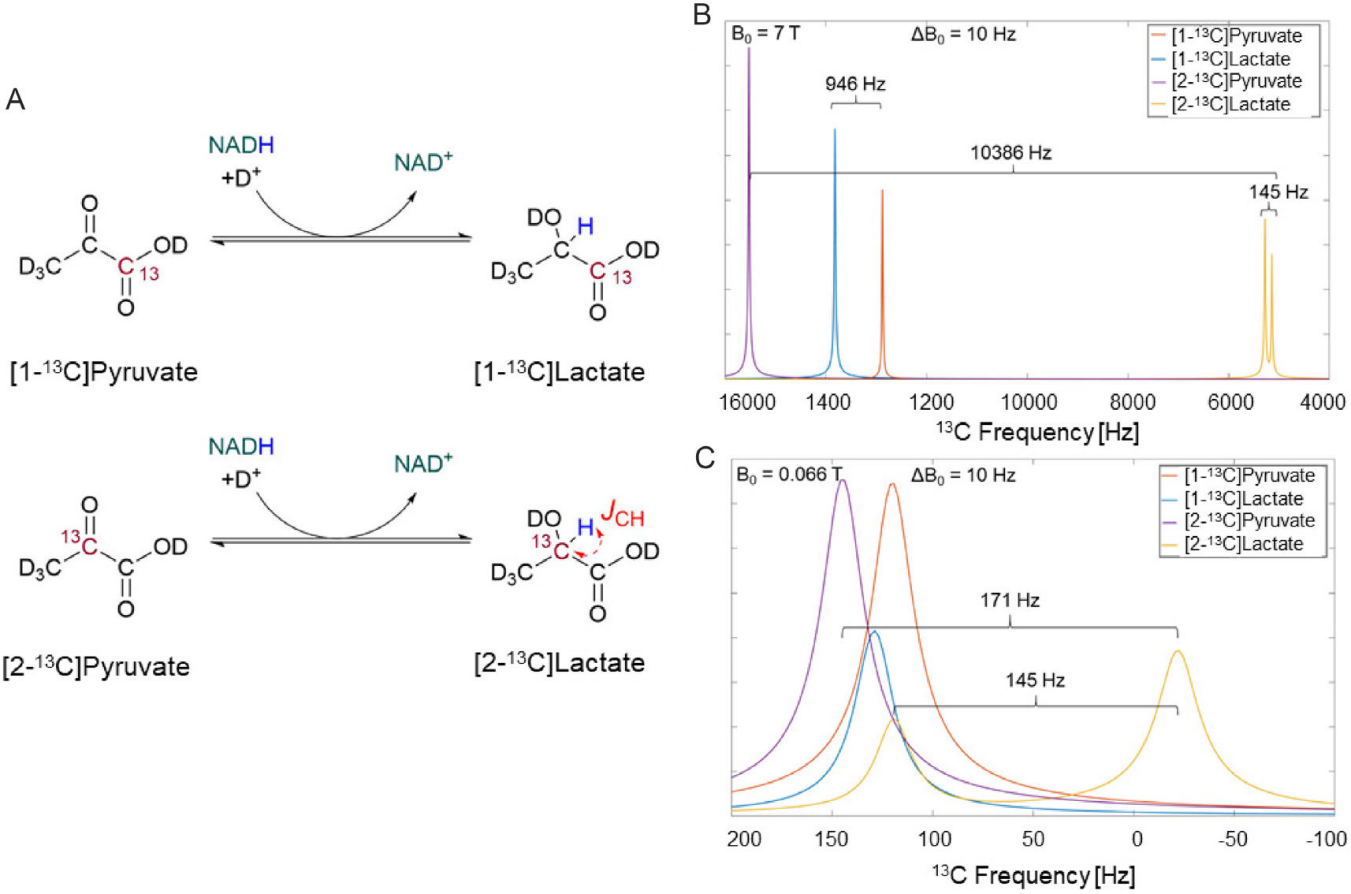
Simulated ^13^C NMR spectra of free [1‐^13^C]pyruvate, [2‐^13^C]pyruvate, [1‐^13^C]lactate, and [2‐^13^C]lactate at high and low fields with an inhomogeneity of 10 Hz. A) Reaction scheme for [1‐^13^C] and [2‐^13^C]pyruvate, the red *J*(C,H) in the [2‐^13^C]lactate denotes the 145 Hz coupling. B) Chemical shift dominated spectra at high magnetic field with *B*_0_ = 7 T. Pyruvate and lactate peaks are separated by several hundreds of Hz given the large chemical shifts. This facilitates the metabolites identification independently of the position of the ^13^C labeling. C) *J*‐coupling dominated spectra at low magnetic field with *B*_0_ = 0.066 T. The minimal chemical shifts make it impossible to differentiate between the [1‐^13^C] labeled pyruvate and lactate. The strong *J*(C,H) coupling of 145 Hz for [2‐^13^C]lactate allows the differentiation of pyruvate and lactate at low and inhomogeneous fields.

Herein, we exploited the strong *J*(C,H) coupling of 145 Hz in [2‐^13^C]lactate to operate on the *J*‐coupling‐dominated regime and, therefore, resolve the pyruvate from the lactate peaks at low magnetic fields. The reaction schemes for [1‐^13^C] and [2‐^13^C]pyruvate to lactate are shown in **Figure** **1**A, and their ^13^C NMR spectra simulations^[^ ^27^ ^]^ at 7 T and 0.066 T with 10 Hz inhomogeneity (Δ*B*_0_) are shown in Figure 1B,C, respectively. The spectra at 7 T are dominated by chemical shifts with several hundreds of Hz separating the pyruvate from lactate peaks for both reaction schemes, while, at low magnetic fields (0.066 T), the spectra are dominated by the *J*‐couplings. With only 10 Hz of inhomogeneity, it is already impossible to differentiate between the [1‐^13^C]pyruvate and [1‐^13^C]lactate. In contrast, the 145 Hz *J*(C,H) coupling in [2‐^13^C]lactate makes it possible to distinguish between [2‐^13^C]pyruvate and [2‐^13^C]lactate at low magnetic and inhomogeneous fields, demonstrating the need of using [2‐^13^C]pyruvate to measure metabolism at low fields.

In this work, we present the full pipeline for measuring metabolism in pancreatic cancer cells by using *J*‐coupling spectroscopy at low magnetic fields. This includes the development and optimization of the following steps: 1) the PHIP‐SAH protocol for providing biologically compatible [2‐^13^C]pyruvate with enough signal enhancements for detecting pyruvate and lactate ^13^C signals in vitro at low fields; 2) the design and in‐house construction of a multinuclear low‐field NMR spectrometer with a four rings design permanent magnet, and *B*_0_ = 0.066 T, a triple‐nuclear RF coil for detecting ^1^H and ^13^C plus a ^23^Na channel incorporated for calibrating ^13^C pulses, and five shimming coils for reaching the spectral resolution required for differentiating pyruvate from lactate; 3) the fingerprint matching method for quantifying the pyruvate and lactate concentration; and 4) the validation of the methodology by measuring synthetic enzymatic pyruvate‐to‐lactate conversion. Finally, we measure metabolism in pancreatic ductal adenocarcinoma (Panc02) cells for three different pyruvate and lactate concentrations.

## 2 Results and Discussion

**Figure 2:**
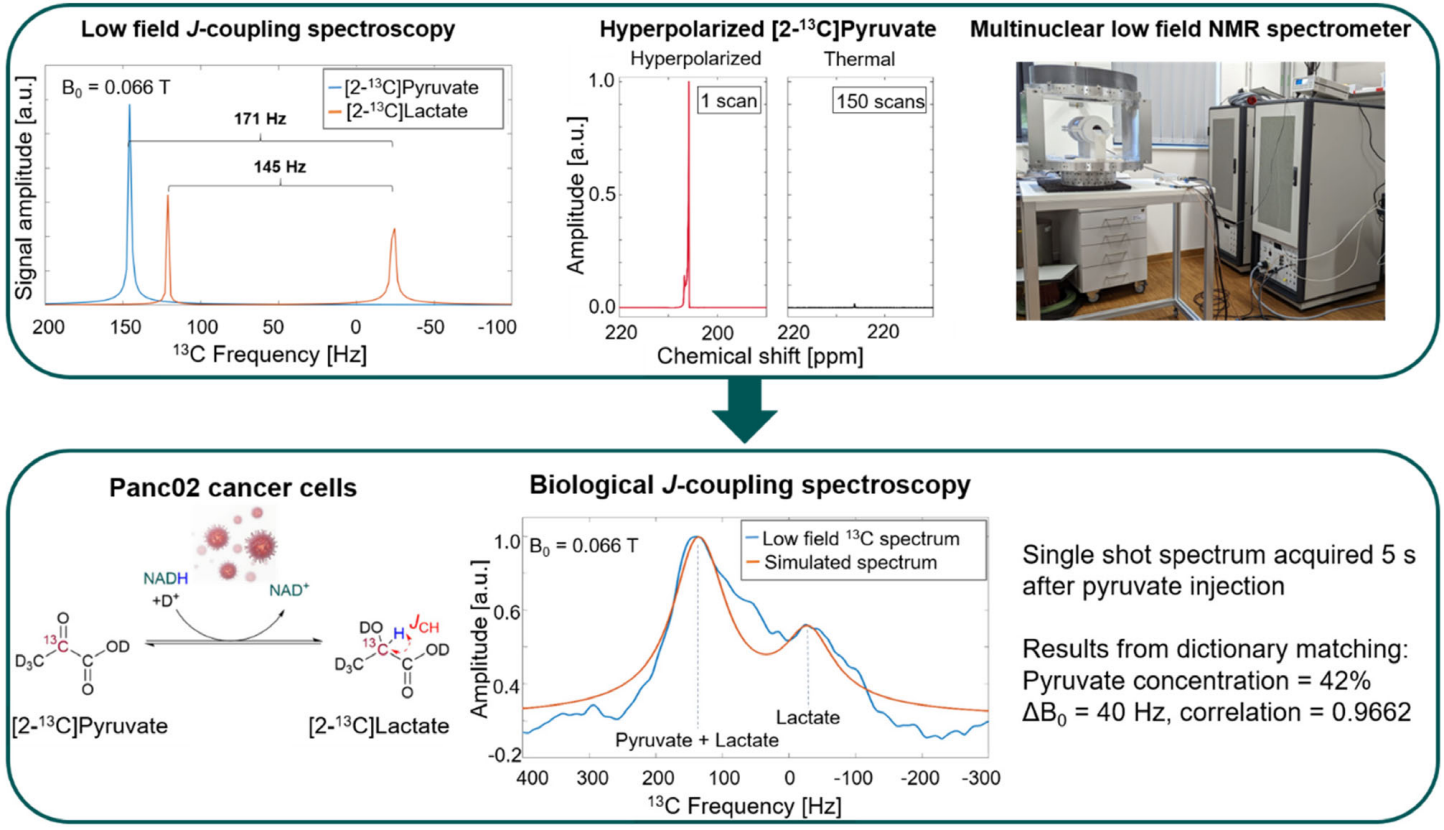
Biological *J*‐coupling spectroscopy. Our team exploited the strong *J*(C,H)‐coupling from [2‐^13^C] to measure the pyruvate‐to‐lactate conversion in cancer cells at low magnetic fields. The simulated pyruvate and lactate spectra at 0.066 T and Δ*B*_0_ = 0 Hz are shown in the top left. This was combined with a PHIP‐SAH‐based HP protocol optimized to provide a biocompatible solution with 20 000‐fold signal enhancement at 7 T (top center image). Additionally, we developed a multinuclear low‐field NMR spectrometer in‐house to be able to acquire the data. This includes the main magnet, shimming, and RF coils (top right image). Finally, we measured the pyruvate and lactate spectra in murine Panc02 cancer cells and calculated the pyruvate concentration and field inhomogeneity through fingerprint matching (bottom image).

**Figure** **2** shows a summary of the main results obtained from our work. This includes the implementation of strong *J*‐couplings for [2‐^13^C]lactate that allow the differentiation with pyruvate at low magnetic fields, the optimization of a PHIP‐SAH HP protocol that provides biocompatible [2‐^13^C]pyruvate with signal enhancements of 20 000‐fold at 7 T that facilitates its detection at low magnetic fields, and the multinuclear low‐field NMR spectrometer developed in‐house (top panel of Figure 2). The bottom panel of Figure 2 shows a pyruvate‐to‐lactate conversion measured at 0.066 T for murine Panc02 cancer cells. The pyruvate + lactate peak can be differentiated from the lactate peak, allowing for concentration quantification. Thus, we implemented a fingerprint matching method to determine the pyruvate concentration and magnetic field homogeneity. The estimated Δ*B*_0_ = 40 Hz describes the observed line broadening. The correlation of 0.9662 shows a good agreement between the simulated and acquired spectra. The following sections describe each step in detail, in addition to the active shimming for reaching the required spectral resolution and the validation of the methodology in LDH enzymatic conversion.

The simulations showed that *J*‐coupling spectroscopy allows the differentiation of [2‐^13^C]pyruvate from lactate at low magnetic fields due to the strong *J*(C,H) coupling of 145 Hz in the [2‐^13^C]lactate. Nonetheless, given the typical inhomogeneities of low‐field scanners based on permanent magnets,^[^ ^28^, ^29^, ^30^ ^]^ the implementation of a shimming strategy is necessary for reaching the minimal required spectral resolution. For our device, the spectral resolution was obtained by implementing just 5 shimming coils (three of them were linear gradients), without the need of previous passive shimming. Nevertheless, further improvement of the shimming will positively impact the SNR and sensitivity of the method.

### 2.1 Biocompatible Hyperpolarized [2‐^13^C]pyruvate

To measure the metabolism at low magnetic fields in biological solutions, the pyruvate needs to fulfil two requirements: the polarization has to be enhanced sufficiently via PHIP to be detectable in the low‐field scanner, and the final pyruvate solution resulting from the HP procedure has to be biocompatible.

**Figure 3:**
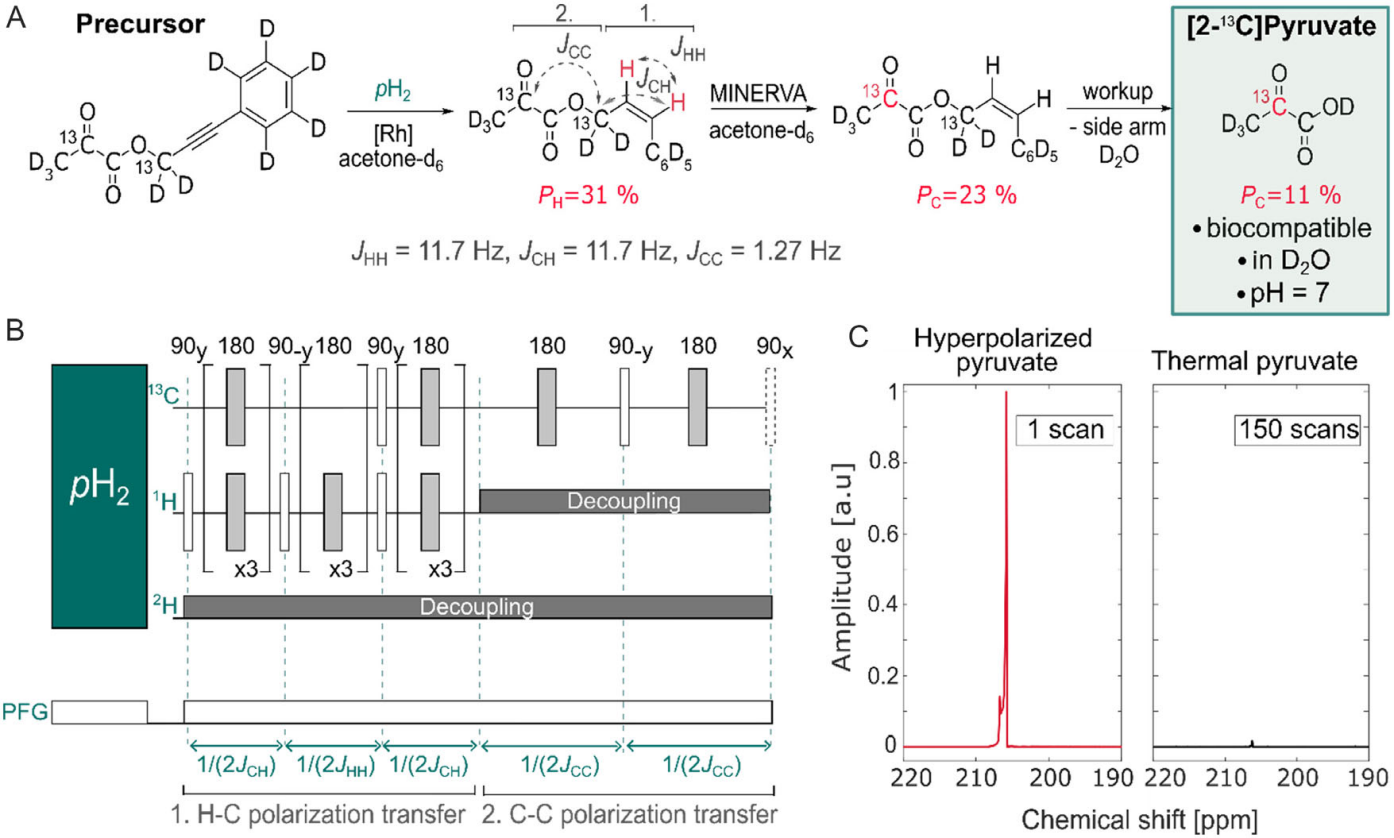
HP of biocompatible [2‐^13^C]pyruvate. A) Reaction scheme of the HP procedure, including hydrogenation polarization transfer and work‐up. B) MINERVA pulse sequence^[^ ^23^, ^30^ ^]^ used for polarization transfer from ^1^H‐spins from *p*H_2_ to 2‐^13^C nucleus of pyruvate. Composite pulses were used as 180° refocusing pulses. C) Red: ^13^C NMR spectrum (7 T) of hyperpolarized [2‐^13^C]pyruvate in biocompatible, aqueous (D_2_O) solution (8 mM, 200 μL), 1 scan. Black: Thermal ^13^C NMR spectrum of non‐hyperpolarized [2‐^13^C]pyruvate in biocompatible, aqueous (D_2_O) solution (8 mM, 200 μL), 150 scans, and intensity was increased by a factor of 10.

**Figure** **3** shows the polarization procedure that our team developed to fulfill both requirements. Catalyzed by a rhodium‐catalyst, the triple bond of the precursor (3‐(phenyl‐*d*_5_)prop‐2‐yn‐1‐yl‐1‐^13^C‐1,1‐*d*_2_ 2‐oxopropanoate‐2‐^13^C‐3,3,3‐*d*_3_, 30 mM) was hydrogenated with hydrogen enriched in the para state leading to a proton polarization of 31 ± 3% (see Figure S1, Supporting Information). A MINERVA pulse sequence^[^ ^24^, ^31^, ^32^ ^]^ was employed to transfer the polarization from the ^1^H‐spins to the targeted 2‐^13^C of the pyruvate in two steps (see Figure 3B). First, the polarization of the ^1^H‐spins is transferred to the ^13^C‐nucleus of the cinnamyl side‐arm. The corresponding delays in the pulse sequence stem from the *J*‐couplings *J*(C,H) and *J*(H,H). In the next step, polarization transfer from the sidearm ^13^C‐nucleus to the 2‐^13^C‐nucleus of the pyruvate moiety using the C‐C coupling (*J*(C,C)) is taking place, reaching a polarization at the 2‐^13^C‐nucleus of 23 ± 2% (see Figure S2, Supporting Information). To make the sequence robust against radiation damping, constant pulsed field gradients (PFG) and refocusing pulses were applied.^[^ ^33^ ^]^

We performed a work‐up procedure to obtain free [2‐^13^C]pyruvate in a biocompatible aqueous solution.^[^ ^24^, ^25^ ^]^ First, we cleaved off the sidearm via saponification. The acetone solvent was removed under reduced pressure, and pH was adjusted to pH = 7 by injecting HEPES buffer (pH = 3) into the solution (see Figure S3, Supporting Information). After filtration to remove the toxic [Rh] catalyst, we obtained hyperpolarized [2‐^13^C]pyruvate in biocompatible, aqueous (D_2_O) solution at concentrations of 8 ± 2 mM. Then, we measured the ^13^C NMR spectra of hyperpolarized pyruvate after work‐up and a thermal spectrum of the same non‐hyperpolarized solution at high magnetic fields to calculate the polarization values obtained (see Figure 3C and Figure S4, Supporting Information). As a result, we measured polarization values of 11 ± 2%. Furthermore, Figure S5 (Supporting Information) shows the thermal measurements for ^13^C concentration quantification, and Figure S6 (Supporting Information) shows three consecutive HP experiments acquired at 0.066 T to demonstrate the repeatability and robustness of our method. Finally, we measured the *T*_1_ time of the 2‐^13^C nucleus in the pyruvate moiety after work‐up at 0.066 T, with *T*_1_ = 45 ± 2 s. Given the short *T*_1_ time of the [2‐^13^C]lactate, it was not possible to measure it, but we expect it to be close to the 5.5 s reported at 0.024 T.^[^ ^27^ ^]^

As far as we are aware, our work presents the PHIP HP protocol with the highest signal enhancement for a biocompatible solution of [2‐^13^C]pyruvate. A comparison of proton polarization (31 ± 3%) and carbon polarization of the 2‐^13^C‐nucleus of the pyruvate moiety before workup (23 ± 2%) shows losses in polarization of about 25% during the MINERVA pulse sequence. This is due to the two‐step polarization transfer, in which not 100% efficient polarization transfer leads to remaining polarization on the ^13^C‐nucleus of the cinnamyl side‐arm. The measured values of both, proton polarization and carbon polarization after work‐up, are in the range of typical polarization values for these kinds of PHIP‐systems.^[^ ^24^ ^]^ Additional 47% of the carbon polarization of the 2‐^13^C‐nucleus of the pyruvate moiety is lost during the work‐up procedure (23 ± 2% before work‐up to 11 ± 2% after work‐up). As shown in Ding et al.,^[^ ^24^ ^]^ this is within the typical losses during work‐up and is mostly related to the *T*_1_ polarization decay of pyruvate during the work‐up. Nevertheless, measured final carbon polarization of 11 ± 2% represents a big improvement with respect to the previously reported value of 3.9 ± 0.4%.^[^ ^24^ ^]^ Bubbling for 9 s instead of 18 s leads to an increase in polarization of the 2‐^13^C‐nucleus of the pyruvate moiety before work‐up of 44% (9 s: 33 ± 3%; 18 s: 23 ± 2%). The high catalyst‐to‐substrate ratio (13 mM/30 mM) enables full hydrogenation already in shorter bubbling times, and polarization decay due to the *T*_1_ time of the nucleus is reduced. For further improvements, the exploration of new precursor molecules that maximize the *J*‐coupling between the ^13^C nucleus in the side‐arm and the [2‐^13^C] site of the carboxylate moiety can play a key role in future studies.

The use of the fully deuterated [2‐^13^C]pyruvate is the simplest spin system for measuring metabolic *J*‐coupling spectroscopy. Nonetheless, the exploration of other probes can result in more rich spectra that potentially increase the sensitivity. For example, the implementation of double‐labeled [1,2‐^13^C_2_]pyruvate can result in a large variety of spectra depending on how the polarization is transferred to the ^13^C (100% to the first, 50% – 50%, or 100% to the second position).^[^ ^31^ ^]^

### 2.2 Multinuclear Low‐Field NMR Spectrometer

**Figure 4:**
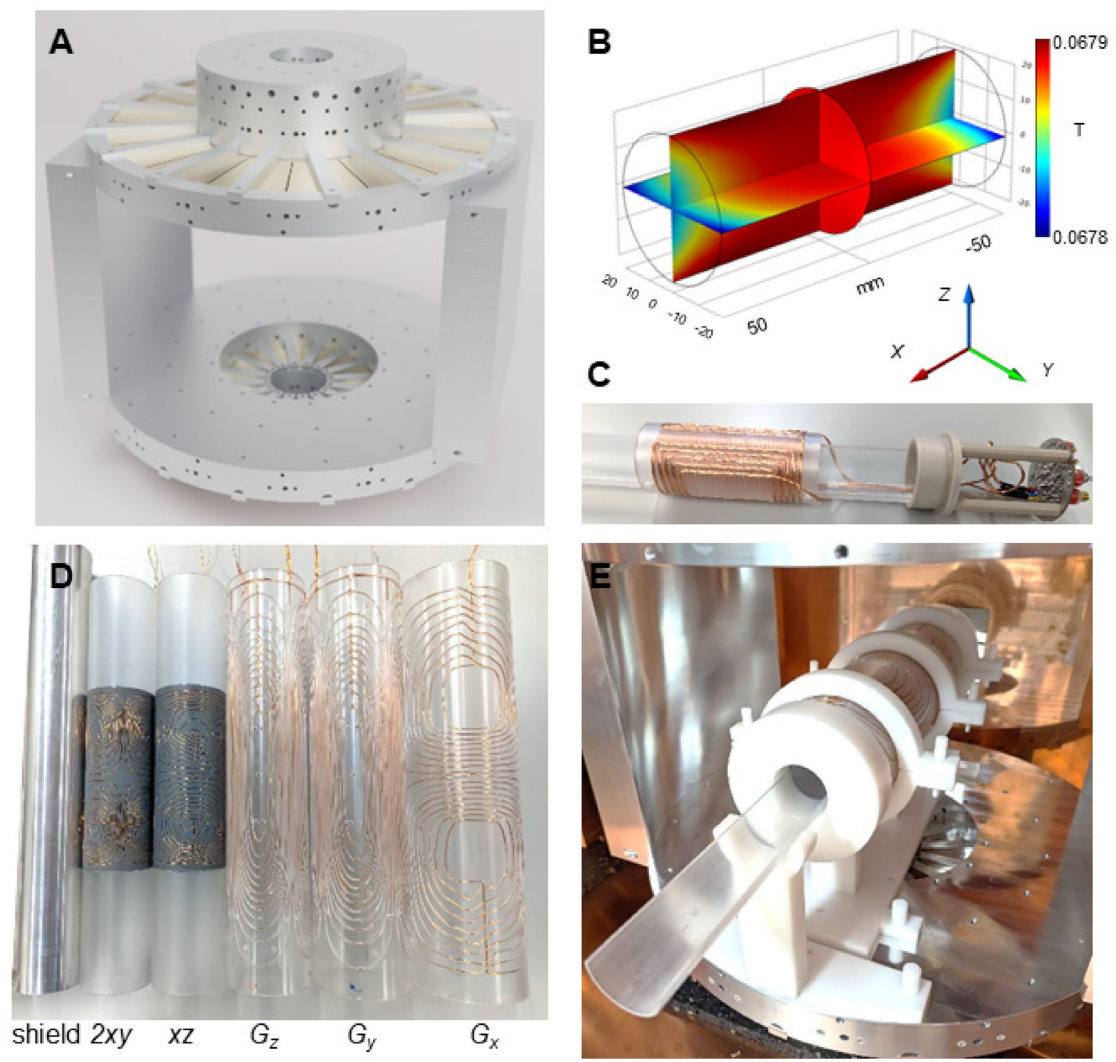
Low‐field multinuclear NMR spectrometer built in‐house. A) Render of the main magnet design. The magnet has a biplanar configuration with a gap of 28 cm, and 64 neodymium magnets constitute it. B) Magnetic field simulation of the main magnet for a cylinder of 100 mm length and 40 mm diameter, and the spatial coordinates axis. C) RF multinuclear coil. The coil is composed of the five‐turn saddle coil that operates at ^1^H frequency and the variable gap solenoid that is used for detection of ^23^Na and ^13^C. The coil has an analogic switch that allows changing between nucleus by selecting different tuning and matching circuits. D) Shield, shimming, and gradient coils. E) Assembly of all the coils inside the magnet.

Our team designed and built in‐house a multinuclear low‐field NMR spectrometer to measure metabolism at low fields. The premises for the magnet design were portability, lightweight, and having room for the incorporation of shimming coils to reach the required spectral resolution for measuring pyruvate‐to‐lactate conversion. As a result, our main magnet consists of a four rings array with axially directed magnetization and mirror symmetry with a 28 cm gap composed by 64 neodymium magnets, as shown in **Figure** **4**A. The frame of the magnet was made from aluminum resulting in a total weight of only 83 kg. The 28 cm gap provides a great versatility to implement several shimming coils and even offers the possibility to plan future in vivo pre‐clinical and clinical studies with the current magnet. With its 83 kg, the magnet is one of the lightest designs currently available.^[^ ^28^, ^29^, ^30^ ^]^ Specific details of the magnet design and construction can be found in the methods section.

Figure 4B shows the *xy* and *xz* planes of the *z* component of the magnetic field simulations implemented in COMSOL Multiphysics 6.1 (Göttingen, Germany) for a field of view defined by a cylinder of 100 mm length and 40 mm diameter placed in the geometrical center of the magnet (see Figure S13, Supporting Information). The simulated field in the center of the magnet is 0.06796 T. The peak‐to‐peak inhomogeneity in the full cylinder volume without the incorporation of the shimming coils is 2796 ppm, with the main inhomogeneity component along the *x* direction.

**Figure 5:**
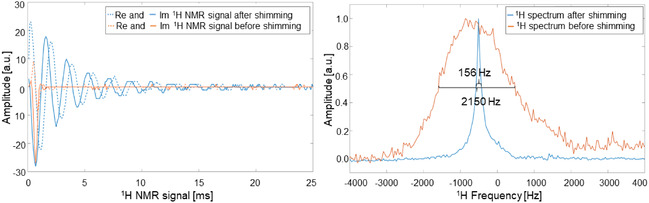
^1^H NMR signal and spectra before and after shimming. The measurements were realized for a 20 mL vial of 25 mm diameter and 57 mm length filled with deionized water. Left: ^1^H NMR signals. Right: ^1^H NMR spectra obtained after implementation of discrete Fourier transform. 2150 and 156 Hz correspond to the full width half maximum of the NMR spectra before and after the shimming implementation, respectively. This is equivalent to 766 and 56 ppm inhomogeneity.

Figure 4C shows our multinuclear RF coil. This triple‐nuclear coil can detect ^1^H, ^23^Na, and ^13^C frequencies. We used the ^1^H channel for adjusting sample positioning and shimming, the ^23^Na for calibrating ^13^C pulses and sequences without the need of enriched ^13^C phantoms or HP, and the ^13^C for detecting the metabolic conversion. The resulting Q‐factors were: 147 ± 7 for ^1^H, 260 ± 40 for ^23^Na, and 240 ± 50 for ^13^C. Then, we detected NMR signals for each nuclei and **Figure** **5** shows ^1^H signals acquired for a 20 mL water phantom, Figure S7 in the Supporting Information shows a ^23^Na signal of a 20 mL phantom with water saturated with sodium chloride after 32 scans, and Figure S8 (Supporting Information) shows ^13^C signals detected for a 5 M ^13^C labeled urea phantom of 20 mL doped with gadolinium after 512 scans and a single scan of 200 μL of a 15 mM solution of hyperpolarized pyruvate in D_2_O. The specifications of the coil design can be found in the methods section.

The 2796 ppm of inhomogeneity obtained from the magnetic field simulations results in a line width of more than 1000 Hz in ^13^C frequency making it impossible to differentiate between pyruvate and lactate at low field. This challenge was addressed by the incorporation of shimming coils to our system to be able to resolve the 171 Hz frequency shift between [2‐^13^C]pyruvate and [2‐^13^C]lactate peaks (see Figure 1B). Therefore, we built five shimming coils for our experiments. The shimming coils, together with the aluminum shield, are shown in Figure 4D. The shield was placed between the RF coil and the shimming coils to improve SNR. For shimming, we constructed a 2*xy* and a *xz* coils given that the simulations showed that *x* is the principal inhomogeneity direction, and three linear gradient coils, that can also be implemented for future spatial encoding experiments. Figure 4E shows the assembly of all the coils inside the magnet. Specifications of the design, construction, and characteristics of the shimming coils are in the methods section, together with the electronics implemented to run the spectrometer.

### 2.3 Achievement of Metabolic Spectroscopic Resolution at Low Field

Once the scanner was assembled, we determined the mean magnetic field and the inhomogeneity of the magnet from a ^1^H NMR spectra of a 20 mL glass vial of 25 mm diameter and 57 mm length filled with deionized water. As *γ*Δ*B*_0_ ≫ 1*/T*_1_ *+ 1/T*_2,_ and the measured NMR spectra were symmetric, the mean magnetic field over the 20 mL volume was determined as the resonance frequency (position of the NMR spectra peak), and the Δ*B*_0_ was estimated as full width half maximum of the spectrum.^[^ ^34^ ^]^ Figure 5 shows ^1^H NMR signals, and their respective spectra acquired for the 20 mL sample before and after shimming. The mean magnetic field for both cases was 0.066 T. The inhomogeneity before and after shimming was 2150 Hz and 156 Hz in ^1^H frequency, which is equivalent to 766 ppm and 56 ppm, respectively. The 0.066 T is slightly lower than the simulated value, while the inhomogeneity is smaller than the simulated given the reduced sample volume. With only five shimming coils, where three of them are linear gradients, it was possible to improve homogeneity by more than an order of magnitude. The 56 ppm is equivalent to 39 Hz in ^13^C frequency, and this is enough resolution to resolve the *J*‐coupling spectra at 0.066 T.

### 2.4 Quantification of Pyruvate Concentration through Fingerprint Matching

**Figure 6:**
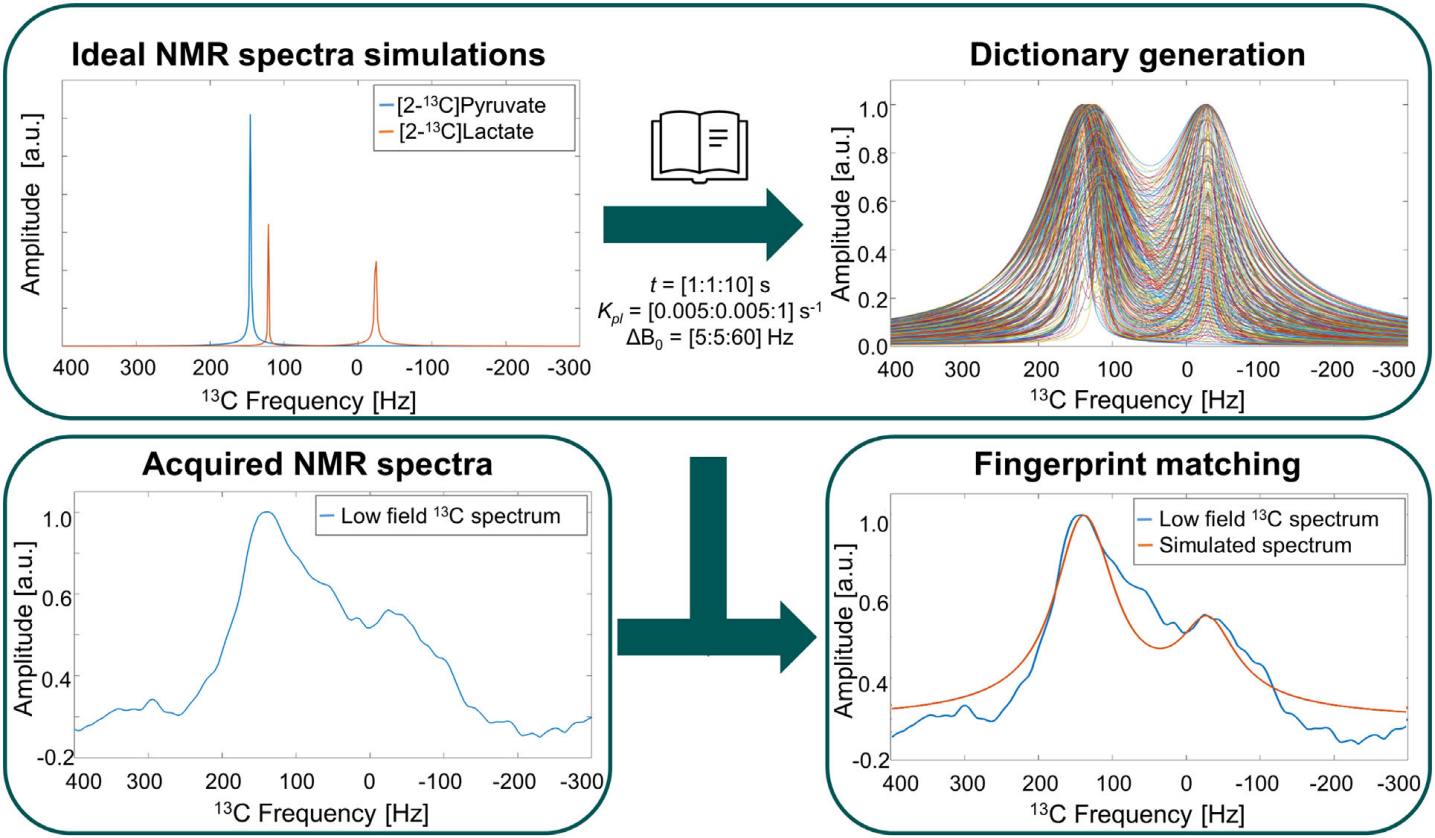
Pipeline of *J*‐coupling spectra fingerprinting. First, the NMR spectra for [2‐^13^C]pyruvate and lactate were simulated in Spinach^[^ ^36^ ^]^ (top left image). Then, the dictionary was generated by mixing the pyruvate and lactate spectra with considering pyruvate to lactate conversion rates $k_{pl}$ ranging from 0.005 to 1.0 s^−1^ by steps of 0.005 s^−1^, evolutions times *t* ranging from 1 to 10 s in steps of 1 s, and ΔB_0_ values ranging from 5 to 60 Hz in steps of 5 Hz (top right image). Finally, the fingerprint matching is implemented to the acquired NMR spectrum (bottom left) by maximizing the correlation with the simulated data and minimizing the peak amplitudes differences (bottom right image).

We implemented a fingerprint approach to determine the pyruvate concentration. Furthermore, the fingerprint also estimates the Δ*B*_0_ of the acquired spectra to take into account variations in the sample volume and positioning between experiments. To consider relaxation and conversion rate effects, a two‐site exchange integral model^[^ ^35^ ^]^ was implemented as follows:

(1) $$M_{py}(t)=M_{py}(0)e^{-(r_{py}+k_{pl})t},\;M_{lac}(t)=\frac{M_{py}(0)k_{pl}}{k_{pl}+r_{py}-r_{lac}}[e^{-r_{lac}t}-e^{-(r_{py}+k_{pl})t}]$$

where $M_{py}$ and $M_{lac}$ are the pyruvate and lactate magnetizations, rpy=1T1,py and rlac=1T1,lac are the corresponding relaxivities, $k_{pl}$ is the conversion rate constant from pyruvate to lactate in $s^{-1}$, and *t* is the evolution time before the spectra acquisition. The back conversion from lactate to pyruvate was neglected, as well as, the conversion from pyruvate or lactate to other metabolites. Additionally, we assumed that at t=0, all the magnetization corresponds to pyruvate, i.e., $M_{lac}\left(0\right)=\;0$. To reduce the number of free parameters, the relaxation values were fixed to values measured in media with phosphate buffered saline (PBS) buffer, with $T_{1,py}=45\;s$ and $T_{1,lac}=5.5\;s$.^[^ ^27^ ^]^ Then, we generated a dictionary containing 24 000 simulated spectra by considering pyruvate to lactate conversion rates $k_{pl}$ ranging from 0.005 s^−1^ to 1.0 s^−1^ by steps of 0.005 s^−1^, evolutions times *t* ranging from 1 to 10 s in steps of 1 s, and Δ*B*_0_ values ranging from 5 Hz to 60 Hz in steps of 5 Hz. The inhomogeneity was expressed in terms of the ^13^C frequency, and the range was determined considering the 39 Hz estimated in the previous sub‐section. The [2‐^13^C]pyruvate and lactate under perfect homogenous field (ideal spectra) were simulated using Spinach with the chemical shifts and *J*‐couplings reported in the materials and methods section.^[^ ^36^ ^]^ Then, the mixed spectra were obtained by adding the simulated spectra calculated from Equation (1) for the corresponding $k_{pl}$ and *t* values. The spectra under inhomogeneity result from the convolution of the mixed spectra and a Lorentzian function with full width half maximum = Δ*B*_0_. The acquired and simulated spectra were normalized and centered using as reference the amplitude and frequency of their highest peak. Before the fingerprinting matching, we selected the simulated spectra that correspond to the evolution time *t* of the experimentally acquired spectra. In this way, we reduce the number of free parameters and the dictionary size to 2400. Finally, we implemented the fingerprint matching with the acquired NMR in two steps. First, the 24 spectra with the highest correlation with the acquired data were selected, which corresponds to the highest 1% spectra from the dictionary. Subsequently, the best spectrum was obtained by minimizing the peak intensity difference between the acquired and simulated data. The two‐steps fingerprint matching strategy showed higher robustness under noisy data, given that the spectrum with the higher correlation does not necessarily yield the best matching.^[^ ^37^ ^]^ **Figure** **6** shows a schematic of the fingerprinting pipeline. It is worth mentioning that, even though the dictionary provides a value for the conversion constant $k_{pl}$, the goal of our work is the measurement pyruvate and lactate concentrations rather than making a quantitative analysis of $k_{pl}$. The calculated $k_{pl}$ is subject to the several approximations mentioned previously, as well as, the uncertainties associated with the evolution time *t*, which can go up to ±2 s in our experiments, and LDH Units or cell numbers measurements. Thus, we back calculated the pyruvate and lactate concentrations by using Equations (1) and relaxation effects. These results are more robust than the obtained for $k_{pl}$ due to all the parameters are contained in one value. Therefore, if one of the parameters is overestimated, the other will compensate for this effect with an underestimation to provide a good matching. For example, if the measured time *t* is shorter than the “real” evolution time, the $k_{pl}$ from the matching will be bigger than the “real” $k_{pl}$.

### 2.5 Enzymatic Pyruvate‐to‐Lactate Conversion at Low Field

**Figure 7:**
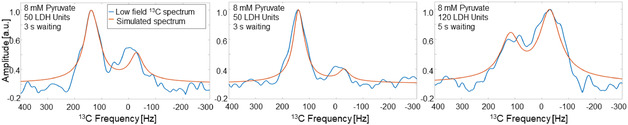
Enzymatic *J*‐coupling pyruvate‐to‐lactate conversion at 0.066 T. The left and middle spectra were measured after 3 s waiting time post injection of 30 mM [2‐^13^C]pyruvate dissolved in 200 μL solution (D_2_O) in a 50 LDH units solution dissolved in HEPES buffer (100 μL,100 mM, pH = 7, in D_2_O) containing NADH (60 mM). The fingerprint matching of the left spectrum results in 51% of pyruvate concentration, 25 Hz of inhomogeneity, and a correlation of 0.9511. The middle spectrum results were 73% pyruvate, 20 Hz inhomogeneity, and 0.9660 correlation. The differences are associated to variations in positioning and timing from injecting the pyruvate and starting the acquisition (±2 s). The right spectrum was measured after 5 s waiting time for a 120 LDH units solution. The fingerprint results were 0% pyruvate, 40 Hz inhomogeneity, and 0.9690 correlation.

**Figure** **7** shows the ^13^C NMR spectra measured after injecting [2‐^13^C]pyruvate into a solution of lactate dehydrogenase (LDH) dissolved in N‐(2‐Hydroxyethyl)piperazine‐N'‐(2‐ethanesulfonic acid) (HEPES) buffer (100 μL, 100 mM, pH = 7, in D_2_O) containing protonated nicotinamide adenine dinucleotide (NADH). The left and central spectra were acquired 3 s after placing the NMR tube inside the scanner for a solution containing 50 units of LDH. The right spectrum was acquired 5 s after injecting the pyruvate in a solution with 120 LDH units. The fingerprint results were: 51% of pyruvate concentration, 25 Hz of inhomogeneity, and a correlation of 0.9511 for the left spectrum, 73% pyruvate, 20 Hz inhomogeneity, and 0.9660 correlation for the central spectrum, and 0% pyruvate, 40 Hz inhomogeneity, and 0.9690 correlation for the right spectrum.

The enzymatic spectra were in good agreement with the simulated spectra obtained from the fingerprint matching (Figure 7). All the correlation values are higher than 0.95. The Δ*B*_0_ values are 25, 20, and 40 Hz from left to right spectra. These values are close or smaller than the 39 Hz Δ*B*_0_ estimated from the half width maximum of the 20 mL phantom. The differences in Δ*B*_0_ between experiments are mainly associated to small volume variations and errors in the positioning. The last point can be minimized by the construction of a sample holder. The difference in pyruvate concentration between the left and middle spectra (both measured for 3 s waiting time and 50 LDH units) is associated with the time variation between injecting the pyruvate and starting the acquisition, which is ±2 s. The spectrum from the right shows a pure lactate spectrum because the 120 LDH units were able to convert all the available pyruvate within just 5 s. As the *T*_1_ of lactate is shorter than the *T*_1_ of pyruvate, the SNR of this last spectrum is lower than the SNR in the previous spectra.

### 2.6 Biological *J‐*Coupling Spectroscopy in Panc02 Cancer Cells at Low Magnetic Field

**Figure 8:**
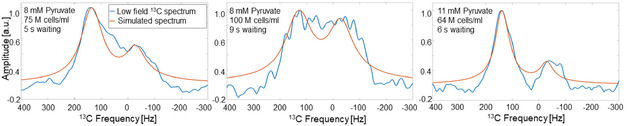
Biological *J*‐coupling pyruvate‐to‐lactate conversion of Panc02 cancer cells at 0.066 T. The left spectrum was measured after 5 s waiting time post injection of 8 mM [2‐^13^C]pyruvate dissolved in 200 μL aqueous solution (D_2_O) in a HEPES buffer (100 μL, 100 mM, pH = 7, in D_2_O) containing 75 M cancer cells/mL. The fingerprint matching of the left spectrum results were 42% of pyruvate concentration, 40 Hz of inhomogeneity, and a correlation of 0.9662. The central spectrum corresponds to an 8 mM pyruvate injection in 100 M cancer cells/mL acquired 9 s after positioning the sample. The results were 14% pyruvate, 50 Hz inhomogeneity, and 0.9580 correlation. The right spectrum was measured after 6 s waiting time for a 11 mM pyruvate solution injected into 64 M cancer cells/mL. The fingerprint results were 55% pyruvate, 25 Hz inhomogeneity, and 0.9600 correlation.

**Figure** **8** shows the ^13^C NMR spectra measured after injecting 200 μL solution with 8 mM/11 mM [2‐^13^C]pyruvate into a solution containing [64–100] M cells/mL murine Panc02 cancer cells. The left spectrum was acquired 5 s after placing the NMR tube inside the scanner for a solution containing 75 M cells mL^−1^ and 8 mM pyruvate solution (concentrations before mixing). The central spectrum was acquired 9 s after placing the NMR tube inside the scanner for a solution containing 100 M cells mL^−1^ and 8 mM pyruvate solution. The right spectrum was acquired 6 s after injecting a 11 mM pyruvate in a solution with 64 M cells mL^−1^. The fingerprint results were: 42% of pyruvate concentration, 40 Hz of inhomogeneity, and a correlation of 0.9662 for the left spectrum, 14% pyruvate, 50 Hz inhomogeneity, and 0.9580 correlation for the central spectrum, and 55% pyruvate, 25 Hz inhomogeneity, and 0.9600 correlation for the right spectrum. The cell viability after the experiments was in all the cases >90%.

The Panc02 cancer cell spectra were in good agreement with the simulated spectra obtained from the fingerprint matching (Figure 8). All the correlation values were higher than 0.95. The Δ*B*_0_ values are 40, 50 , and 25 Hz from left to right spectra. As for the enzymatic data, the variations in Δ*B*_0_ are mainly attributed to different positioning. Also consistent with the enzymatic spectra, the spectrum with the lowest pyruvate concentration exhibits the lower SNR. As expected, longer waiting times result in lower pyruvate concentration and higher pyruvate injection coupled with a lower number of cells results in higher pyruvate concentration.

## 3 Conclusion

In conclusion, our work introduces the implementation of *J*‐coupling spectroscopy in biological samples at low magnetic fields. The differentiation of pyruvate and lactate demonstrates that it is possible to study metabolism in milli‐Tesla fields. Low‐field NMR spectroscopy offers a promising approach for studying biological systems in their native environments, providing insights into molecular behaviors inaccessible through high‐field NMR. Its application in in vitro studies is particularly advantageous, enabling cost‐effective analysis of biological samples. Future studies will include the development of in vivo applications, including portable devices for imaging metabolism and spectroscopy in living organisms.

## 4 Experimental Section

4.1

4.1.1

##### NMR Simulations

The ^13^C NMR spectra for [1‐^13^C]pyruvate, [1‐^13^C]lactate, [2‐^13^C]pyruvate, and [2‐^13^C]lactate were simulated at high (7 T) and low fields (0.066 T) with a *B*_0_ inhomogeneity (Δ*B*_0_) of 10 Hz. We simulated the spectra with Δ*B*_0 _= 0 Hz in Spinach^[^ ^36^ ^]^ using the chemical shifts ([1‐^13^C]pyruvate 170.5 ppm, [1‐^13^C]lactate 183.0 ppm for ^13^C and 1.4 ppm for ^1^H, [2‐^13^C]pyruvate 203.4 ppm, [2‐^13^C]lactate 66.5 ppm for ^13^C and 1.4 ppm for ^1^H) and *J*‐couplings ([1‐^13^C]lactate *J*(C,H) = 4 Hz, [2‐^13^C]lactate *J*(C,H) = 145 Hz) previously measured at our high‐field spectrometer. The Δ*B*_0_ effects were modeled as the convolution of the NMR spectra under homogeneous conditions with a Lorentzian function with half width maximum of 10 Hz. The Δ*B*_0_ = 10 Hz was chosen as a lower bound value for the inhomogeneity, considering that the typical inhomogeneity of low‐field and portable devices typically ranges between 100 and 2000 ppm.^[^ ^28^, ^29^, ^30^ ^]^ For the dictionary matching generation, the Δ*B*_0_ values range from 5 to 60 Hz by steps of 5 Hz. The sweep and number of points were 62 000 and 32 516, respectively, for all the simulations. Then, we adjusted the bandwidth and number of points to match exactly the acquired spectra.

##### HP Procedure

For the HP, we prepared a solution of catalyst [1,4‐Bis(diphenylphosphino)butane](1,5‐cyclooctadiene)rhodium(I) tetrafluoroborate (13 mM, Sigma‐Aldrich 79255‐71‐3) and precursor 3‐(phenyl‐*d*_5_)prop‐2‐yn‐1‐yl‐1‐^13^C‐1,1‐*d*_2_ 2‐oxopropanoate‐2‐^13^C‐3,3,3‐*d*_3_ (220 μl, 30 mM, in acetone‐d6) in a 5 mm NMR tube. The precursor was synthesized, as described in ref. [27]. The solution was degassed with N_2_ for 1 min and transferred into a 7 T Bruker (Avance III) spectrometer at 330 K. In the spectrometer, para hydrogen (95% *para*‐enrichment, generated from a home‐built, automated set‐up) was bubbled through the solution for 18 s at 7 bar with a flow rate of 0.04 ± 0.01 L/min. A MINERVA pulse sequence^[^ ^24^, ^31^ ^]^ (see Figure 3B, further described below) was used to transfer the polarization from the protons to the 2‐^13^C of the pyruvate.

After pressure release, we performed a work‐up to release free pyruvate in biocompatible solutions, as described previously.^[^ ^24^, ^25^ ^]^ Briefly, 0.1 mL of a base solution, consisting of Na_2_CO_3_ (75 mM), EDTA (20 mM), and sodium ascorbate (1 mM) in D_2_O, was added to the hydrogenated solution to cleave the ester bond and scavenge potential radicals.^[^ ^38^ ^]^ Outside the spectrometer close to a 400 mT magnet plate, the acetone solvent was removed from the reaction mixture under reduced pressure for 7 s in a water bath at 79 °C. Then, the pH was adjusted by adding 0.1 mL of HEPES buffer (100 mM, pH = 3, in D_2_O), and the aqueous solution was filtered through a syringe filter (pore size 1.0 μm, Whatman) to remove the catalyst. The filtrate containing pyruvate 8 ± 2 mM, with a ^13^C polarization of 11 ± 2%, in D_2_O buffer (pH = 7) was obtained in a syringe and was either put again in an NMR tube into the spectrometer to measure final polarizations or transferred to the low‐field scanner. Non‐hyperpolarized cinnamyl alcohol (side‐arm) remained in the final solution at concentrations of ≈3 mM (80 μg). In several pharmacologic investigations, cinnamyl alcohol showed no cytotoxic effects in vitro.^[^ ^39^, ^40^ ^]^

##### MINERVA Pulse Sequence

The employed MINERVA pulse sequence (Figure 3B) is based on the pulse sequence for four‐spin systems with two parahydrogen ^1^H and two ^13^C nuclear spins introduced by Stevanato et al.^[^ ^31^ ^]^ It was adapted to the present system by using the corresponding coupling constants (*J*(H,H) = 11.7 Hz, *J*(C,H) = 11.7 Hz, *J*(C,C) = 1.27 Hz) and offset (63 Hz). The pulse sequence is divided into two blocks (see Figure 3B). In the first block, the polarization of the two ^1^H spins stemming from parahydrogen is converted into magnetization on the ^13^C‐spin of the side arm. The second block is used to transfer the magnetization from the sidearm ^13^C to the 2‐^13^C nucleus of the pyruvate. As samples with precursor concentrations higher than 15 mM suffer from polarization loss due to radiation damping,^[^ ^41^, ^42^ ^]^ constant PFG were applied during parahydrogen bubbling (0.4 mT m^−1^, corresponding to 0.05% at BRUKER Avance III gradient system) and pulse sequence (0.4 mT m^−1^, corresponding to 0.05% at BRUKER Avance III gradient system) according to Korchak et al.^[^ ^33^ ^]^ Furthermore, the transverse magnetization in the first transfer process (block 1) is refocused three times utilizing 180° pulses to correct for diffusion effects introduced by the gradients and dephasing due to magnetic field inhomogeneities. Refocusing 180° pulses were composite pulses 90_*x*_180_*y*_90_*x*_ (indicated with gray color).

##### Multinuclear Low‐Field NMR Scanner Built In‐House: Magnet

We implemented an optimization method based on the zonal harmonic description of the magnetic field for designing our magnet.^[^ ^43^ ^]^ The dimensions of the four rings are the result of nonlinear optimization launched over a space of zonal momenta of the magnetic field, given the symmetry of four‐ring design.^[^ ^43^ ^]^ The final dimensions and main characteristics of the magnet can be found in the results section. Sixty‐four individual segments with two different shapes (32 magnets each) compose the magnet: shape 1 is a 22‐degree segment of the ring with radius 47.9 mm, outer radius 100.0 and 71.0 mm height; shape 2 is a 22‐degree segment of the ring with inner radius 121.7 mm, outer radius 225.0 and 25.0 mm height. The positioning of the individual magnets in the aluminum structure required the construction of a specific tool and the labor of one to two people. The details for the assembling are described in the Supporting Information, Figure S9–S11 show renders of the magnet frame, and Figure S12, a schematic of the tool used for the assembly.

##### Multinuclear Low‐Field NMR Scanner Built In‐House: RF Coil

The coil design consists of a concentric saddle and solenoid coils geometrically decoupled. The saddle coil has 5 loops with a diameter of 46 mm and a length of 90 mm. The solenoid has 35 loops with a gap of 3 mm for the 15 center loops, a gap of 2.5 mm for the 5 consecutive loops, and 2 mm for the 5 external loops to improve the B_1_ homogeneity. The diameter of the solenoid is 40 mm and the length 90 mm. Both coils were wired with Litz wires with 60 filaments to improve SNR.^[^ ^44^ ^]^ The saddle coil was dedicated to ^1^H detection, while the solenoid for X‐nuclei detection. For selecting the resonance frequencies of the different nuclei, an analogic switch was incorporated to the RLC circuit to select different matching and tuning capacitor. In addition to the switch, variable capacitors were incorporated for fine adjustment of tuning and matching for each resonance frequency.

##### Multinuclear Low‐Field NMR Scanner Built In‐House: Shimming Coils

Five cylindrical shimming coils were design and built in‐house. The G_*y*_ and G_*z*_ coils were designed based on a target field method reported in the literature,^[^ ^45^ ^]^ while for the G_*x*_, *xy*, and *2xz* the open source CoilGen software was used.^[^ ^46^ ^]^ CoilGen offers several improvements because it already considers the size of the wires and the crossing between the path. The wire paths for the linear coils were machined with a computerized numerical control (CNC) device in acrylic glass (PMMA), while the *xy* and *2xz* coils were 3D printed with Formlabs Grey Resin V4 (Somerville, United States). Then, the wires were manually placed through the paths and glue with superglue. The external diameters of the linear coils were G_*x*_= 100 mm, G_*y*_ = 90 mm, and G_*z*_ = 80 mm. The G_*x*_, G_*y*_, and G_*z*_ showed a gradient efficiency of 1.05 mT m^−1 ^A^−1^, 2.25 mT m^−1 ^A^−1^, and 2.79 mT m^−1 ^A^−1^, respectively. The 2*xy* and *xz* had an external diameter of 64 and 68 mm.

##### Multinuclear Low‐Field NMR Scanner Built In‐House: Electronics

The scanner is controlled by Tecmag Bluestone LF2 MRI console (Houston, United States) with two Tomco (Adelaide, Australia) RF amplifiers. The linear gradient coils are connected to three AE Techron 2110 amplifiers (Elkhart, United States) and the *xy* and *2xz* to a 6‐channel shimming power supply Resonance Research Inc (Billerica, United States).

##### Enzymatic Preparation

LDH (50 units type XI from rabbit muscle, lyophilized powder or 120 units, type II from rabbit muscle, ammonium sulfate suspension) was dissolved in 100 μL HEPES buffer (100 mM, pH = 7, in D_2_O) containing NADH (60 mM). The solution was transferred to a 5 mm NMR tube and maintained at 37 °C before PHIP measurement. All the experiments were acquired after injecting a biocompatible 200 μL aqueous (D_2_O) solution with hyperpolarized [2‐^13^C]pyruvate, at concentrations of 8 ± 2 mM.

##### Cells Preparation

Murine pancreatic ductal adenocarcinoma cells (Panc02), derived from C57BL/6 mice after chemical induction of pancreatic tumors by 3‐methylcholanthrene (MCA),^[^ ^47^ ^]^ were kindly provided by Professor Stine Pedersen (Section for Cell Biology and Physiology, Department of Biology, Faculty of Science, University of Copenhagen, Denmark). They were cultured in Dulbecco's Modified Eagle's Medium (DMEM) in the presence of sodium pyruvate, L‐glutamine, and D‐Glucose (catalog # 1995065, Gibco, USA), and supplemented with 10% fetal bovine serum (FBS) and 1% penicillin/streptomycin at 37 °C, 6% CO_2_ in a humidified incubator. For the experiments, the cells (64–100 M) were collected in a falcon tube and pelleted by centrifugation (1.000 × g) at room temperature. After removal of the supernatant, the cells were suspended in the supplemented DMEM (160 μL). The cell slurry was transferred into a 5 mm NMR tube and maintained at 37 °C before the PHIP‐SAH experiment. Cell viability was measured before and after the PHIP experiment via trypan blue staining.

##### Spectral Measurements at Low‐Field Scanner

The enzymatic/cell solution was maintained in a ThermoMixer (Eppendorf) next to the low‐field scanner at 37 °C. The HP procedure was performed at the high‐field spectrometer as described above. The transfer from the polarizer to the low‐field spectrometer took ≈10 s. During transport, the syringe containing the sample was placed into a 1 T magnet for minimizing *T*_1_ decay. Then, the pyruvate solution was injected into the tube with the enzymatic/cell solution, the mixture was placed into the low‐field spectrometer, and spectra were recorded after 3–9 s. All the spectra were acquired using a spin‐echo sequence with an echo time of 6 ms, acquisition delay of 0.8 ms, and acquisition windows of 102 ms with 1024 points. The acquisition parameters were optimized to maximize SNR, and the spin‐echo sequence was used to avoid the long dead time in the free induction decay (FID) experiment, given the long coil ringing at these ultra‐low frequencies (≈704 kHz). Then, the data were reconstructed using a factor 2 zero filling and implementing a line broadening exponential filter of 10 Hz for all the acquisitions.

## Conflict of Interest

The authors declare no conflict of interest.

## Author Contributions

**Gonzalo G. Rodriguez**, **Sergey Korchak**, **Jan Felger,** and **Oscar Sucre** designed and constructed the hardware; **Gonzalo G. Rodriguez** developed the software; **Gonzalo G. Rodriguez**, **Sergey Korchak**, and **Charlotte von Petersdorff‐Campen** optimized the hyperpolarization protocol; **Gonzalo G. Rodriguez**, **Charlotte von Petersdorff‐Campen**, and **Lisa M. Fries** realized the experiments; **Gonzalo G. Rodriguez**, **Charlotte von Petersdorff‐Campen**, and **Stefan Glöggler** wrote the paper; **Andrea Markus**, **Maria D. Santi**, and **Josef Elsasser** prepared the cells. **Ruhuai Mei** synthetized the precursor. **Frauke Alves** supervised the in vitro experiments. **Stefan Glöggler** conceived the project. All authors revised the manuscript.

## Supporting information

Supplementary Material

## Data Availability

The data that support the findings of this study are available from the corresponding author upon reasonable request.
