# Supplementary material for: Biological J‐Coupling Spectroscopy at Low Magnetic Field
Source: Small Sci. 2025 Jul 31;5(11):2500268. doi: 10.1002/smsc.202500268 (PMC12622443; doi:10.1002/smsc.202500268)
Supplement: Supplementary file 1 — Supplementary Material [file SMSC-5-2500268-s001.pdf]

Supplementary material

Polarization Calculations

The signal enhancements ε_{hyp} at high magnetic fields were calculated using the following equations[24]:

$$\varepsilon_{\text{hyp}} = \frac{I_{\text{hyp}}}{I_{\text{th}}} \cdot \frac{NS_{\text{th}}}{NS_{\text{hyp}}} \cdot \frac{\sin(\theta_{\text{th}})}{\sin(\theta_{\text{hyp}})} \quad (1)$$

I_{hyp} and I_{th} are the integrals extracted from the hyperpolarized and thermal spectra, *NS* is the respective number of averages used to acquire the spectra. Θ is the RF pulse flip angle (90° for all experiments).

The polarization of the hyperpolarized moiety P_{hyp} can then be calculated by multiplying the signal enhancement ε_{hyp} with the thermal polarization P_{th}.

$$P_{\text{hyp}} = \varepsilon_{\text{hyp}} \cdot P_{\text{th}} \quad (2)$$

The thermal polarization P_{th} is definied as follows:

$$P_{\text{th}} = \tanh\left(\frac{h\, w_0}{2k_{\text{B}}T}\right),\ w_0 = -\gamma B_0 \quad (3)$$

Where γ is the gyromagnetic ratio, B_0 the applied magnetic field and T the temperature, along with the Planck h and Boltzmann k_{B} constants.

Proton polarization

The proton polarizations were determined with 3 measurements at 7 T using the following protocol[47]. Before the pH_2 experiment, a ^{1}H-NMR spectrum and a ^{13}C-NMR spectrum were recorded. Then, pH_2 was bubbled through the solutions of the precursor (3-(phenyl-d_5)prop-2-yn-1-yl-1-^{13}C-1,1-d_2 2-oxopropanoate-2-^{13}C-3,3,3-d_3) and catalyst in acetone-d_6 (200 µL) for 18 s and proton spectra were recorded with a $\theta = 45°$ pulse (deuterium and carbon decoupling). The recorded spectra are shown in Figure S1.

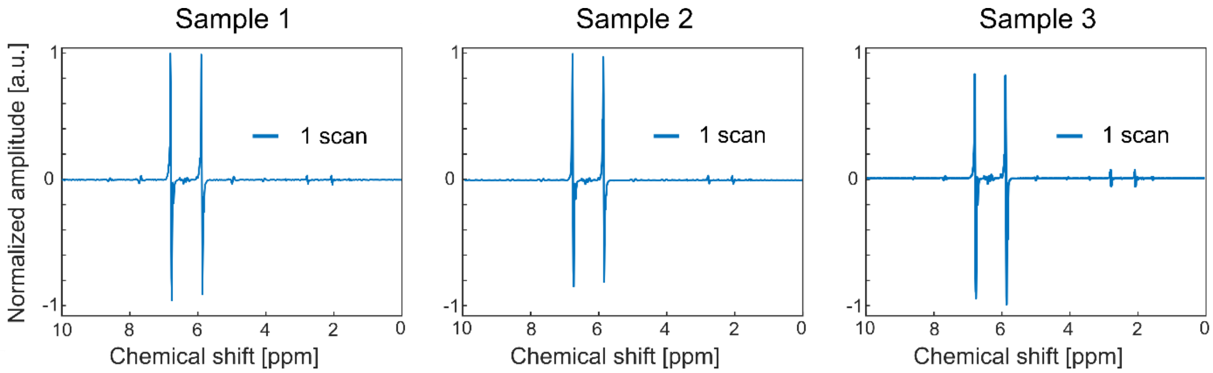

Fig. S1. Repeatability of proton polarization directly after bubbling at high magnetic fields. Three consecutive ^{1}H-NMR (300 MHz) acquisitions (1 scan) directly after bubbling with pH$_2$ through the precursor/catalyst solution in acetone-d_6 (200 µL) acquired at 7 T with a $\theta = 45°$ pulse. The baseline was adjusted.

We measured the following integrals for each of the signals. For each sample the average integral was determined, by adding the absolute values of all the measured integrals and dividing it by the number of protons (2).

Sample 1: I(proton, 6.8 ppm) = 156,869,709 (integrated within 6.8 – 6.9 ppm) and
-179,992,693 (integrated within 6,5 - 6,7 ppm);
I(proton, 5.9 ppm) = 162,380,948 (integrated within 5.9 – 6.1 ppm) and
-161,185,228 (integrated within 5.6- 5.8 ppm);
$I(\text{proton, average})$ = 330,214,289.

Sample 2: I(proton, 6.8 ppm) = 144,730,953 (integrated within 6.8 – 7.0 ppm) and
-163,734,346 (integrated within 6,5 - 6,8 ppm);
I(proton, 5.9 ppm) = 144,798,492 (integrated within 5.9 – 6.1 ppm) and
-142,251,382 (integrated within 5.6- 5.9 ppm);
$I(\text{proton, average})$ = 297,757,586.

Sample 3: I(proton, 6.8 ppm) = 142,940,596 (integrated within 6.8 – 6.9 ppm) and
-163,544,414 (integrated within 6,5 - 6,7 ppm);
I(proton, 5.9 ppm) = 145,608,928 (integrated within 5.9 – 6.1 ppm) and
-147,428,681 (integrated within 5.6- 5.8 ppm);
$I(\text{proton, average})$ =299,761,309.

Because of the specific characteristics of parahydrogen, the observed magnetization is modulated by the product of $\sin\theta \cdot \cos\theta$. Thus, a 45° pulse results in the largest observable signals. To convert the hyperpolarization integrals from a 45° pulse to a 90° a conversion factor of $f = 2$ was used.

$$I_{\text{hyp}} = f \cdot I(\text{proton, average}) \quad (4)$$

Since the hydrogenated sample is highly volatile, no thermal proton spectra after the experiment could be measured to obtain a value for I_{th}. Instead, we used the ^{1}H-NMR spectrum and ^{13}C-NMR spectrum recorded before the hydrogenation to estimate values for the thermal proton integral.

First, the actual precursor concentration needed to be determined. Since the precursor is fully deuterated, only the ^{13}C-NMR spectrum could be used for this purpose. The integral of the ^{13}C peak from the pyruvate moiety $I_{\mathrm{C,s}}$ in the precursor molecule measured with $NS_{\mathrm{C,S}} = 1$ scan was compared to a reference ^{13}C-NMR spectrum of free [2-^{13}C]pyruvic acid, measured under exactly the same conditions (330 K, reference in 200 µL acetone-d$_6$). The concentration of the sample c_{s} could be determined using equation 5.

$$c_{\mathrm{s}} = \frac{I_{\mathrm{C,s}}}{I_{\mathrm{C,ref}}} \cdot \frac{NS_{\mathrm{C,ref}}}{NS_{\mathrm{C,S}}} \cdot c_{\mathrm{C,ref}} \qquad (5)$$

With $I_{\mathrm{C,ref}}$ being the integral and $NS_{\mathrm{C,ref}}$ the number of scans of the ^{13}C reference spectrum and $c_{\mathrm{C,ref}}$ the concentration of the reference ^{13}C sample.

Then, we used the actual precursor concentration c_{s} to estimate the thermal proton integral I_{th}. To achieve this, a proton reference was needed and we used the catalyst in the exact sample for it. Its signals were recorded in the ^{1}H-NMR spectrum measured before the experiment with $NS_{\mathrm{H,ref}} = 1$ scan. To determine the proton reference integral $I_{\mathrm{H,ref}}$ the signals of 24 protons on the catalyst were integrated and normed to one proton. The corresponding thermal proton integral of our sample could then be calculated using equation 6.

$$I_{\mathrm{th}} = \frac{I_{\mathrm{H,ref}}}{c_{\mathrm{H,ref}}} \cdot \frac{c_s}{NS_{\mathrm{H,ref}}} \qquad (6)$$

Table 1 shows all the values for the estimated proton integral of the precursor and the values measured to calculate it.

Table1: Values for calculating the thermal integrals I_{th} over the equations 5 and 6 and the corresponding results for I_{th}.

	$I_{\mathrm{C,s}}$	$NS_{\mathrm{C,ref}}$	$c_{\mathrm{C,ref}}$ /mM	$I_{\mathrm{C,ref}}$	$I_{\mathrm{H,ref}}$	$c_{\mathrm{H,ref}}$ / mM	I_{th}
Sample 1	34,983	5	447	3,618,880	28,013	14,4	41,822
Sample 2	37,719	5	447	3,618,880	27,702	14,4	44,594
Sample 3	36,736	5	447	3,618,880	27,049	14,4	42,408

Using equations 1,2, 3 and 4 and the values for I_{th} from table 1, the proton polarizations were calculated to be:

Sample 1: $P_{\mathrm{hyp,H}} = 34\%$,

Sample 2: $P_{\mathrm{hyp,H}} = 29\%$,

Sample 3: $P_{\mathrm{hyp,H}} = 31\%$.

This method for determination of proton polarizations is based on the assumption that all the unsaturated precursor in the sample is hydrogenated and that a selective hydrogenation to the

double bond in the side-arm is taking place. The determined values for proton polarization are within the range of typical polarizations as measured before [25].

Hyperpolarized ^{13}C spectra detected at 7 T before work-up

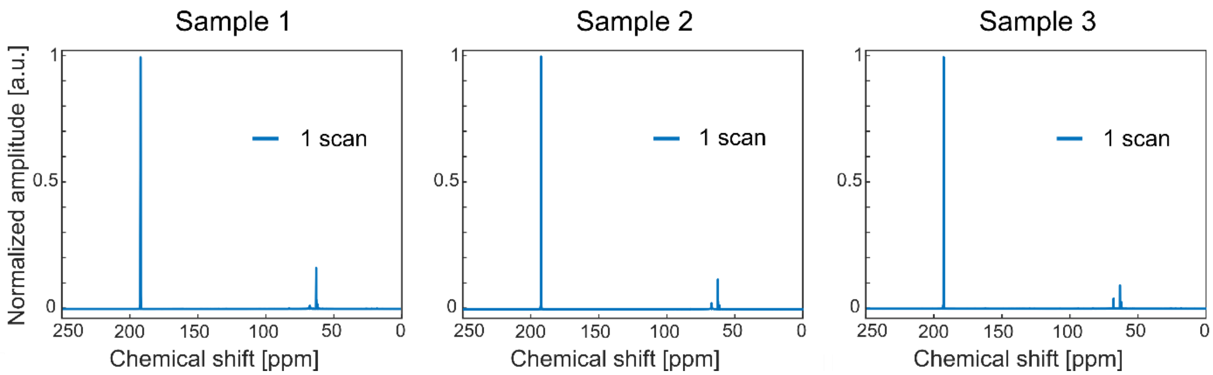

Fig. S2. Repeatability of ^{13}C signal before work-up acquired at high magnetic fields. Three consecutive ^{13}C-NMR (75 MHz) spectra (1 scan) acquired after bubbling with pH$_2$ through the precursor/catalyst solution in acetone-d_6 (200 µL) and polarization transfer to targeted ^{13}C before work-up at 7 T. The spectra were phase corrected and the baseline was adjusted.

The following integrals were observed:

Sample 1: *I*(pyruvate) =1,458,170,215 (integrated within 191.3 – 193.3 ppm);

Sample 2: *I*(pyruvate) = 1,630461445 (integrated within 191.3 – 193.6 ppm);

Sample 3: *I*(pyruvate) = 1,557,615,967 (integrated within 192.2 – 194.4 ppm).

The signal at 62 ppm can be assigned to the ^{13}C nucleus of the side-arm, which is hyperpolarized due to inefficient polarization transfer between the two ^{13}C nuclei.

Due to high volatility of the hydrogenated product, no thermal ^{13}C spectra of the hydrogenated products could be measured. So, again we resorted to measure the thermal integrals before bubbling and polarization, assuming that full and selective hydrogenation of all of the unsaturated precursor was taking place. The following integrals of the corresponding thermal spectra I_{th} (not shown) were obtained after 1 scan.

Sample 1: *I*(pyruvate) = 35,960;

Sample 2: *I*(pyruvate) = 41,385;

Sample 3: *I*(pyruvate) = 34,789.

Using the equations 1,2,3 and the listed values for the integrals, the following carbon polarizations before work-up could be calculated:

Sample 1: P_{hyp} = 22%,

Sample 2: $P_{hyp} = 22\%$,

Sample 3: $P_{hyp} = 24\%$.

pH determination after workup

pH values of the solutions after workup were determined using pH indication paper.

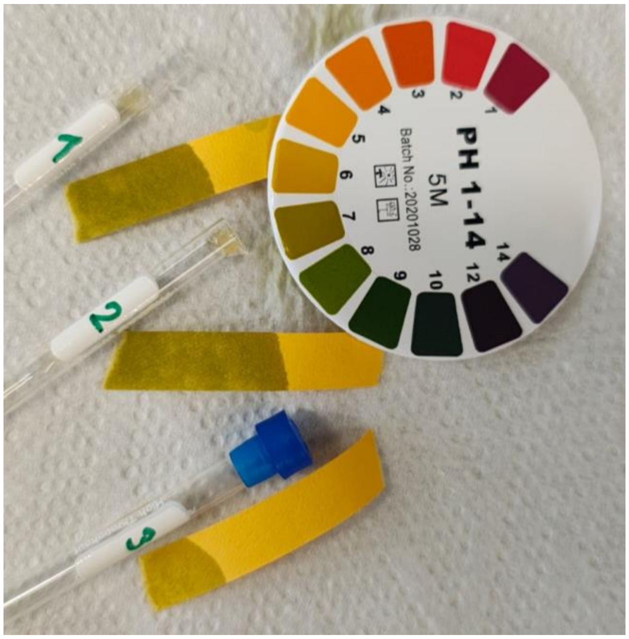

Fig. S3. pH determination after workup. Three consecutive pH determinations via pH indication paper of hyperpolarized pyruvate solution after workup.

Hyperpolarized [2-^{13}C]pyruvate detected at 7 T after work-up

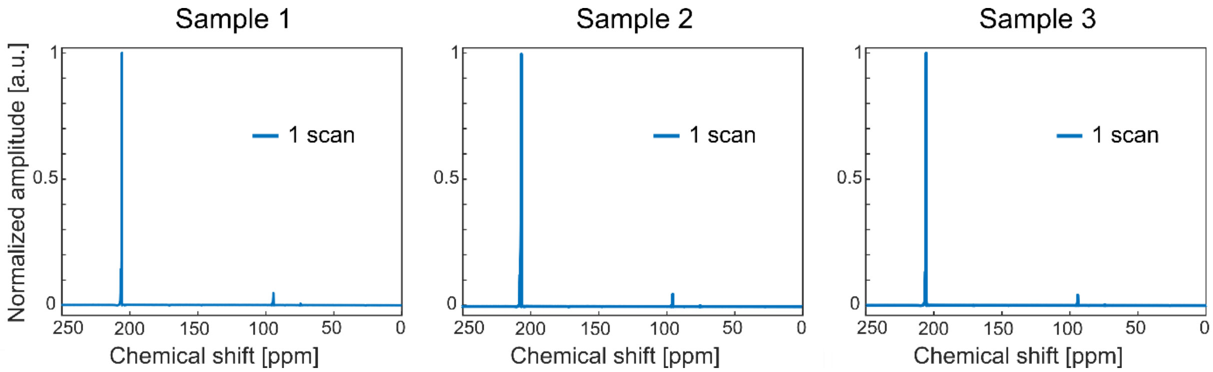

Fig. S4. Repeatability of hyperpolarized pyruvate signal after work-up acquired at high magnetic fields. Three consecutive ^{13}C-NMR (75 MHz) acquisitions of a 200 µL solution with hyperpolarized [2-^{13}C]pyruvate acquired at 7 T. The spectra were phase corrected and the baseline was adjusted.

The following integrals, resulting from integration from 204 – 209 ppm, were observed:

Sample 1: *I*(pyruvate) = 225,975,475;

Sample 2: *I*(pyruvate) = 245,336,722;

Sample 3: *I*(pyruvate) = 288,149,304.

The peak at 95 ppm can be assigned to pyruvate hydrate.

Thermal spectra after work-up

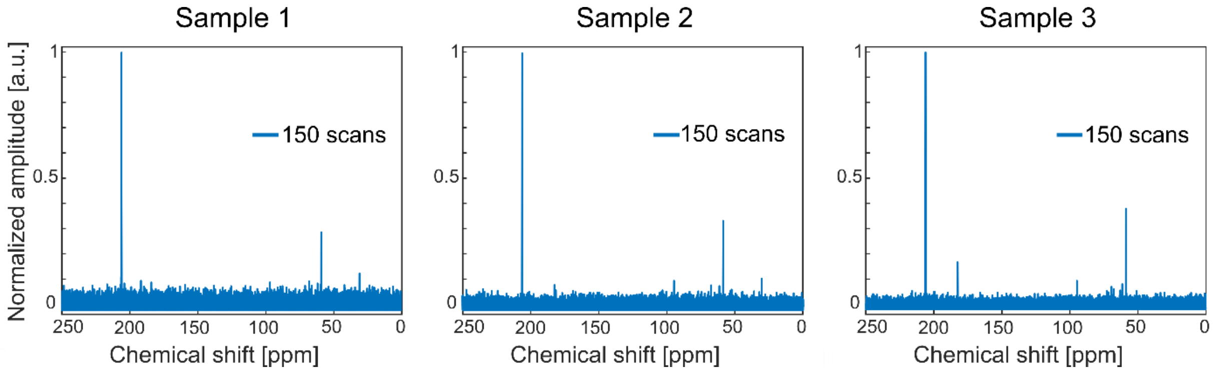

Fig. S5. Thermal ^{13}C-NMR spectra of pyruvate solutions after work-up (150 scans). Three consecutive ^{13}C-NMR acquisitions of a 200 µL pyruvate solution after work-up acquired at 7 T. The spectra were phase and baseline corrected.

Concentrations were calculated using reference samples of [2-^{13}C]pyruvic acid. The following integrals and concentrations were observed:

Sample 1: *I*(pyruvate) = 1,611,023 (integrated within 206.05 – 206.68 ppm); *c*(pyruvate) = 7 mM,

I(cinnamyl alcohol) = 719,701 (integrated within 58.42 –59.16 ppm); *c*(cinnamyl alcohol) = 2.9 mM,

Sample 2: *I*(pyruvate) = 2,126,873 (integrated within 206.02 – 206.48 ppm); *c*(pyruvate) = 9 mM,

I(cinnamyl alcohol) =654,641 (integrated within 58.57 – 58.85 ppm); *c*(cinnamyl alcohol) = 3.0 mM,

Sample 3: *I*(pyruvate) =2,172,826 (integrated within 206.01 – 206.41 ppm); *c*(pyruvate) = 8 mM;

I(cinnamyl alcohol) = 779,576 (integrated within 58.61 – 58.85 ppm); *c*(cinnamyl alcohol) = 3.1 mM.

The following polarization values after work-up were calculated sing equations 1-3 and the corresponding hyperpolarized and thermal integrals.

Sample 1: $P_{hyp} = 12\%$,

Sample 2: $P_{hyp} = 9\%$,

Sample 3: $P_{hyp} = 11\%$.

Hyperpolarized [2-^{13}C]pyruvate detected at 0.066 T

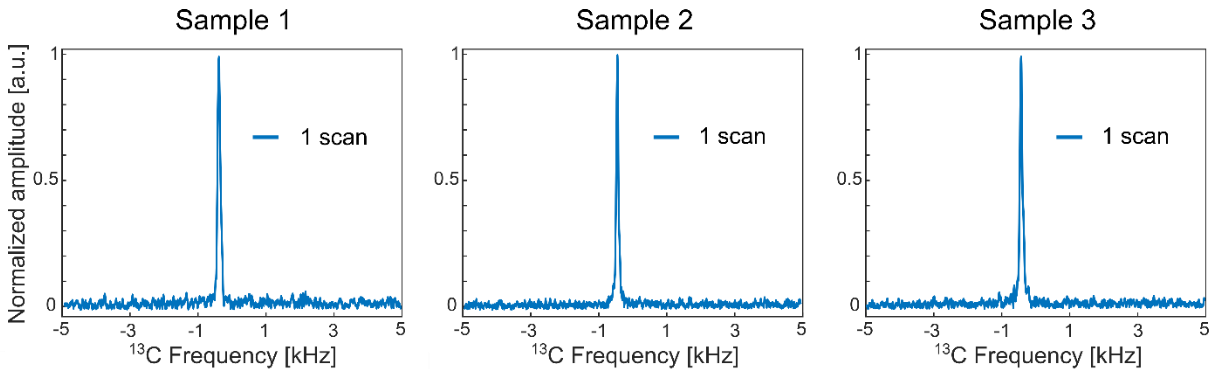

Fig. S6. Repeatability of hyperpolarized pyruvate signal acquired at low magnetic fields. Three consecutive ^{13}C acquisitions of a 200 µL solution with 8 ± 2 mM pyruvate concentration acquired at 0.066 T.

^{23}Na signal acquired in multi-nuclear low field NMR spectrometer

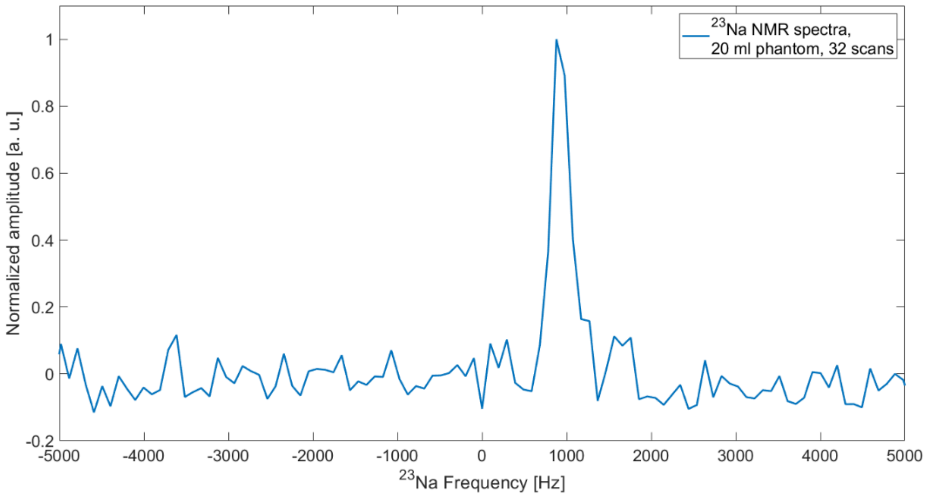

Figure S7. Normalized ^{23}Na NMR spectra acquired at 0.066 T. 32 scans of 20 mL of water saturated with sodium chloride.

^{13}C signals acquired in multi-nuclear low-field NMR spectrometer

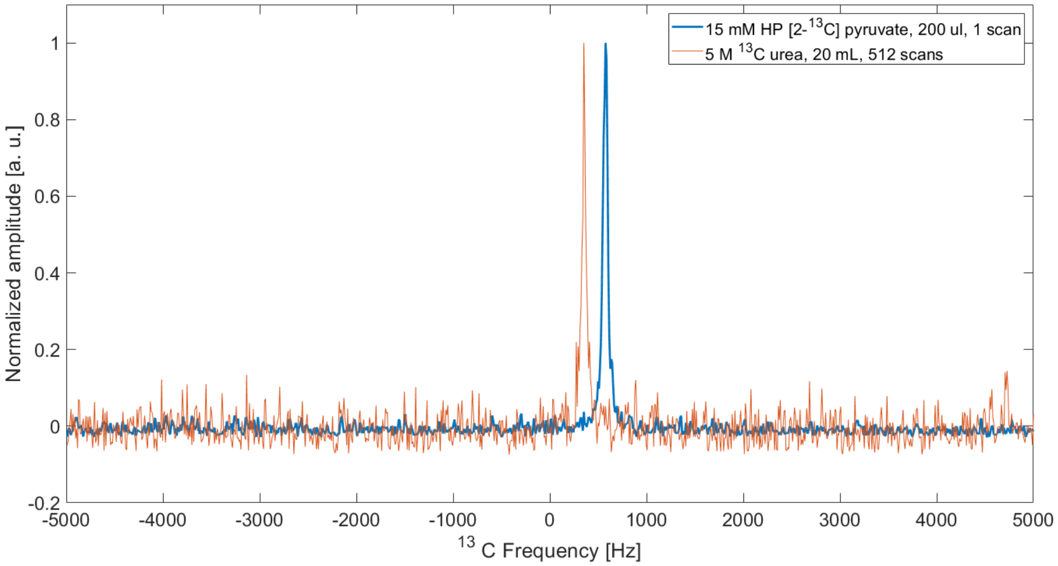

Fig. S8. Normalized ^{13}C NMR spectra acquired at 0.066 T. Blue line, spectra from 1 scan of 200 µL of 15 mM hyperpolarized ^{13}C pyruvate in D_2O; orange, spectra from 512 scans of 5M ^{13}C labeled urea sample doped with gadolinium.

Magnet assembling

The magnet consists of two large outer rings and two small inner rings. To assemble the large ring segments 3D-printed plastic pieces were designed to form a tunnel for installing each segment. Two steel rods were preventing the already installed segments from shifting and keeping the place for a new segment. An aluminum foot was used to press a magnet segment down to the desired place against the magnetic forces. The mechanical manual pressing machine is not shown. Once the new segment was in between the already installed segments, the steel rods were removed and the new segment was pressed to the final position. The foot has a hole allowing a fixing bar to be inserted after installation of the segment. The similar principle was used to install the small segments in the inner ring. Fig. S7 shows the render of the main magnet, Fig. S8 shows different views of the aluminum rings frame, Fig. S9 shows different views of the aluminum columns, and Fig. S10 the tools used for the magnet assembly.

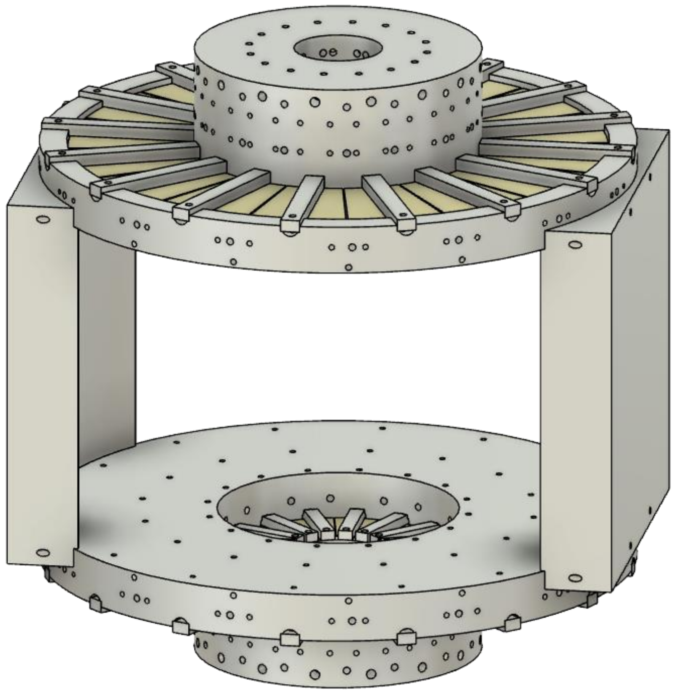

Fig. S9. Main magnet render design. The magnet frame was built from aluminum and the permanent magnet rings are composed of 64 neodymium magnets segments.

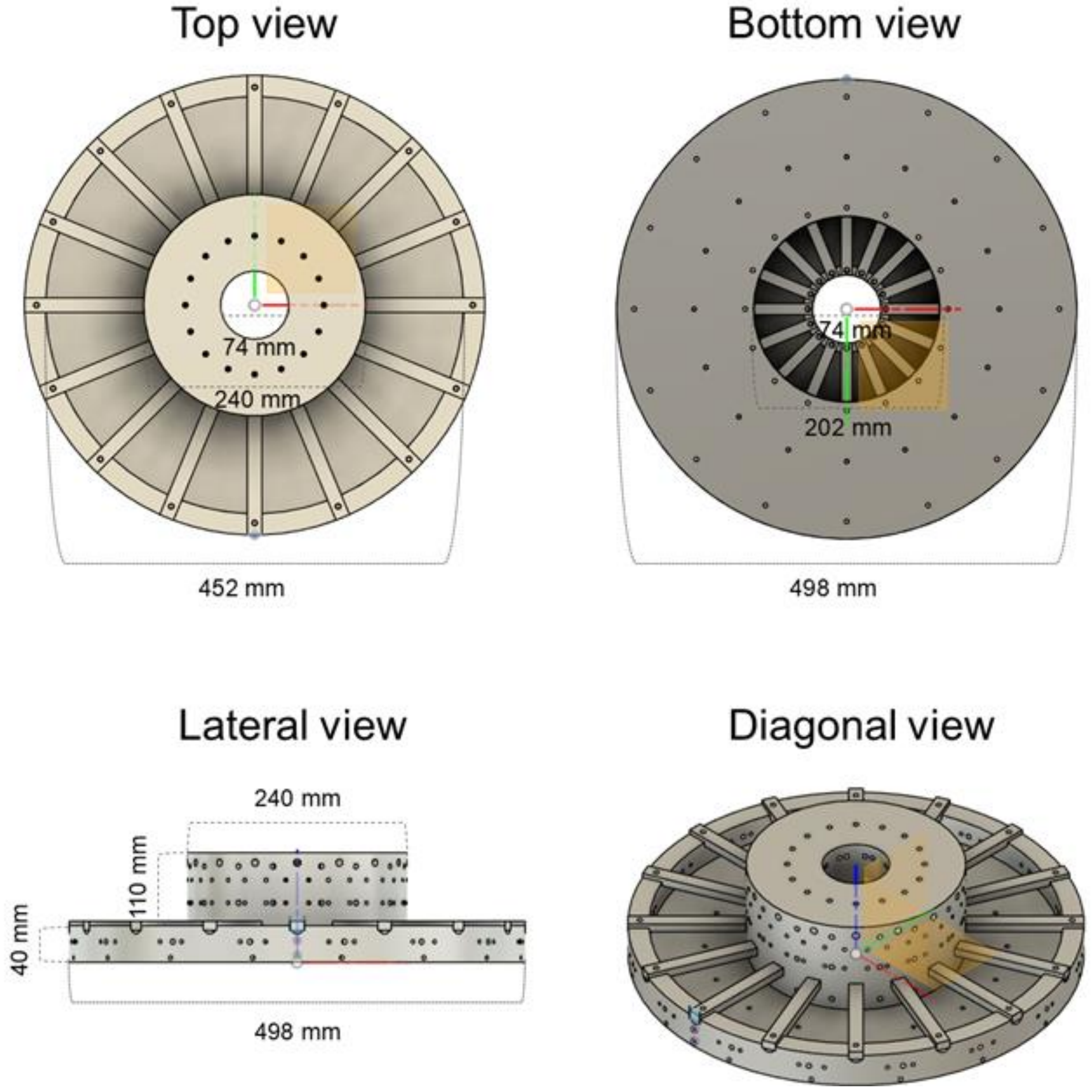

Fig. S10. Aluminum rings structure render. Top, bottom, lateral, and diagonal views of the render of the aluminum rings frame used for the magnets positioning is shown together with the diameters and heights of every part.

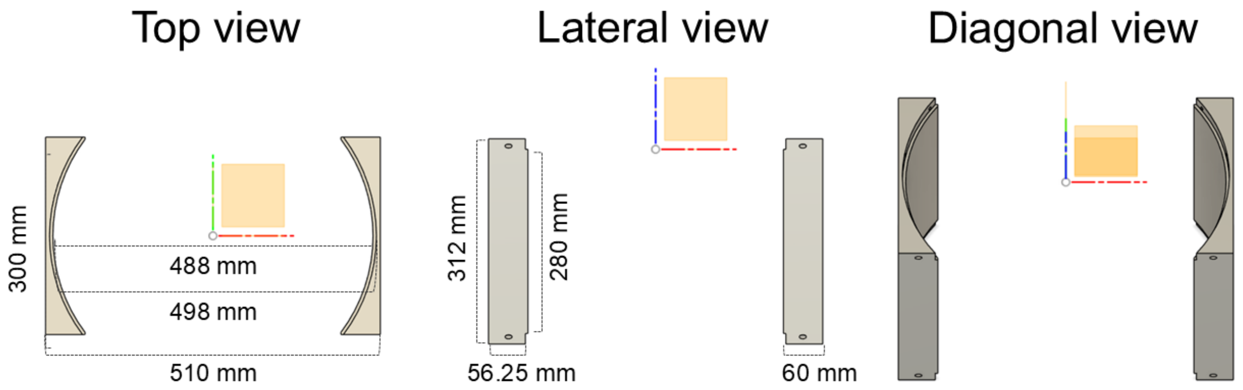

Fig. S11. Aluminum columns structure render. Top, lateral, and diagonal views of the render of the aluminum columns used as frame for the magnets positioning is shown together with the diameters and heights of every part.

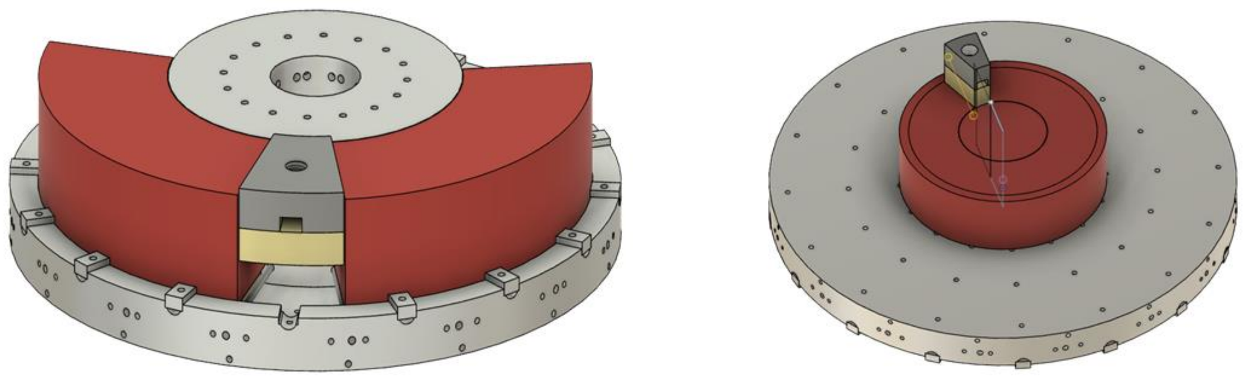

Fig. S12. Magnet assembling. Red, 3D printed plastic structures to guide the magnet positioning. Dark gray are tools used for positioning the large (left) and small (right) magnet ring segments.

Magnet simulations

Figure S13 shows the COMSOL design used for the magnet simulations.

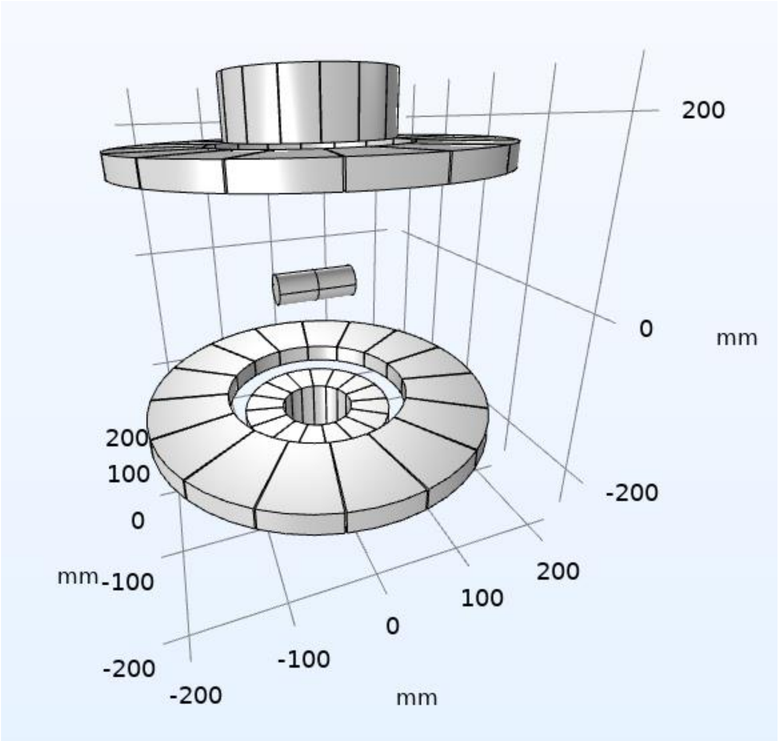

Fig. S13. Magnet simulations. Four magnet rings composed of segments used in COMSOL simulations. The cylindrical region 100 mm long and 40 mm diameter in the center was optimized for best field homogeneity. In the result the segments have following shapes: small segments are 22 degree segment of the ring with radius 47.9 mm, outer radius 100.0 mm and 71.0 mm height large segments are 22 degree segment of the ring with inner radius 121.7 mm, outer radius 225.0 mm and 25.0 mm height.
